# Supplementary material for: M1BP cooperates with CP190 to activate transcription at TAD borders and promote chromatin insulator activity
Source: Nat Commun. 2021 Jul 7;12:4170. doi: 10.1038/s41467-021-24407-y (PMC8263732; doi:10.1038/s41467-021-24407-y)
Supplement: Supplementary file 1 — Supplementary Information [file 41467_2021_24407_MOESM1_ESM.pdf]

SUPPLEMENTARY INFORMATION

M1BP cooperates with CP190 to activate transcription at TAD borders and promote chromatin insulator activity

Supplementary Figure 1

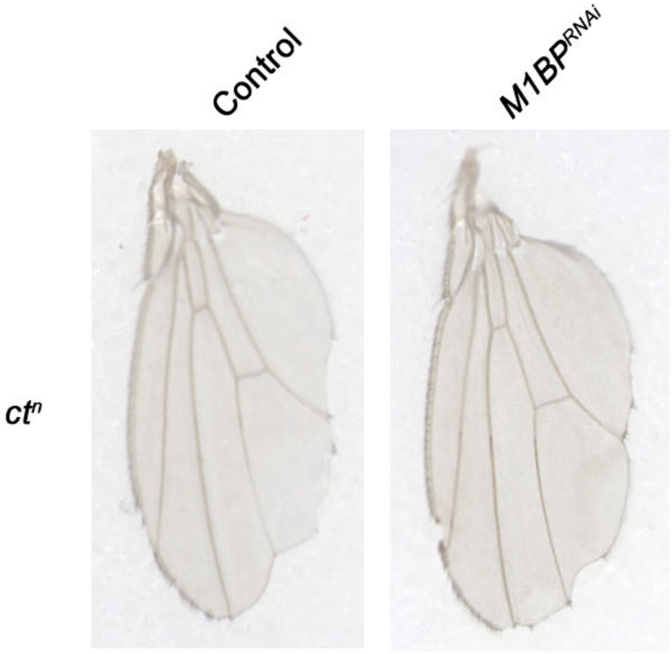

Supplementary Figure 1. Related to Figure 2. Depletion of M1BP does not affect *gypsy*-independent ct^n phenotype.

Knockdown of *M1BP* does not affect phenotype of the *gypsy*-independent ct^n allele. Wild type (left) and $M1BP^{RNAi}$ lines (right) driven by *Ser-Gal4* display similar wing margin phenotypes for the *gypsy*-independent ct^n loss-of function allele. n = 50 flies were assayed for each genotype, and a representative wing is shown.

Supplementary Figure 2

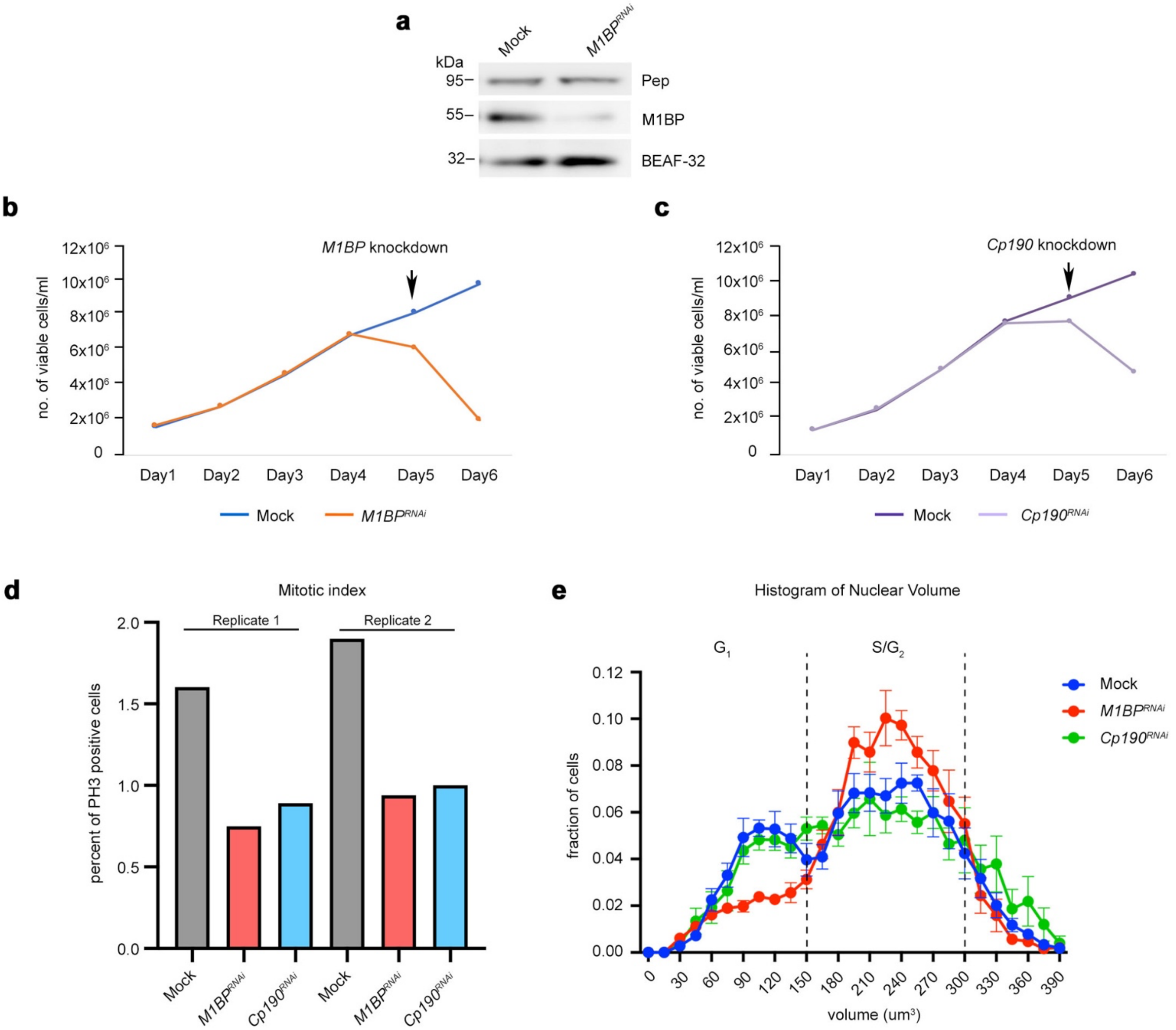

Supplementary Figure 2. Related to Figure 3 and 6. Functional analysis of M1BP and CP190.

a Western blotting of total lysates from Kc control Mock and *M1BP* knockdown cells showing knockdown efficiency of *M1BP* after 5 days of dsRNA transfection and no effect on protein levels of BEAF-32. Pep is used as loading control. Western blotting was performed two times with similar results. **b** Viable cell counts of Mock or *M1BP* knockdown cells at indicated times after dsRNA transfection in Kc cells. Data represented is the average of two biological replicates. **c** Viable cell counts of Mock or *Cp190* knockdown cells at indicated times after dsRNA transfection in Kc cells. Data represented is the average of two biological replicates. **d** PH3ser10 positive cells were counted in viable (Tubulin positive) Mock, *M1BP* knockdown and *Cp190* knockdown cells after 5 days of knockdown after transfection. Bar graphs show the mean for two biological replicates, where n = 888, 1454, or 1482 cells (Mock, *M1BP* knockdown, and *Cp190* knockdown, respectively; rep1) and n = 1104, 1171, and 1010 cells (Mock, *M1BP* knockdown, and *Cp190* knockdown, respectively; rep2). **e** Graph representing cell cycle stage in Mock, *M1BP* knockdown, and *Cp190* knockdown based on nuclear volume. Data represented as mean ± SE of the average of four biological replicates.

Supplementary Figure 3

a

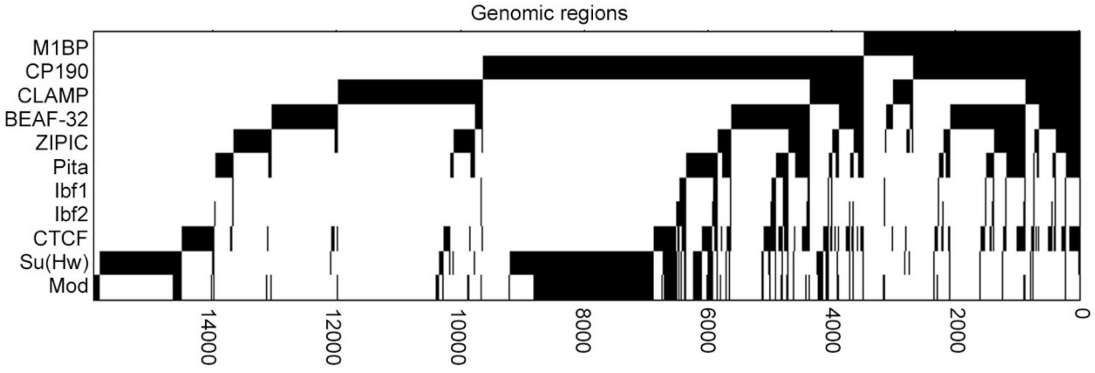

b

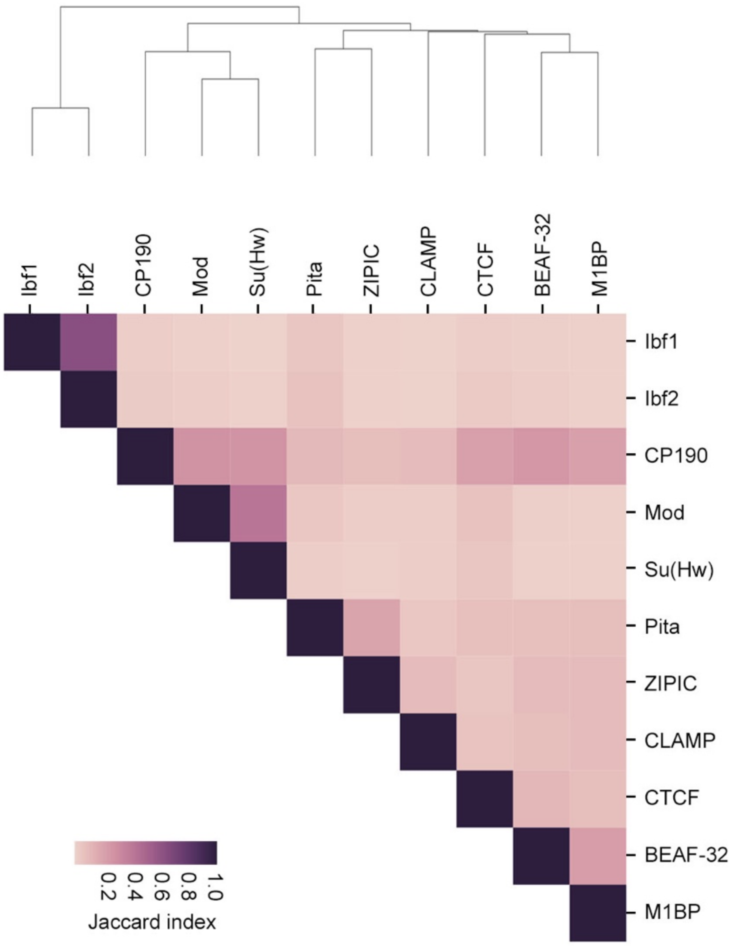

Supplementary Figure 3. Related to Figure 3. M1BP co-localizes with CP190 and other factors.

a Binary heat map shows comparison of binding of M1BP, CP190, CLAMP, BEAF-32, Su(Hw), Mod(mdg4)67.2, and CTCF in Kc cells and ZIPIC, Pita, Ibf1, and Ibf2 in S2 cells. A black mark in a row indicates presence of the particular factor at that genomic region.

b Jaccard heatmap shows heatmap of pairwise comparisons of colocalization of M1BP with *gypsy* components and other factors.

Supplementary Figure 4

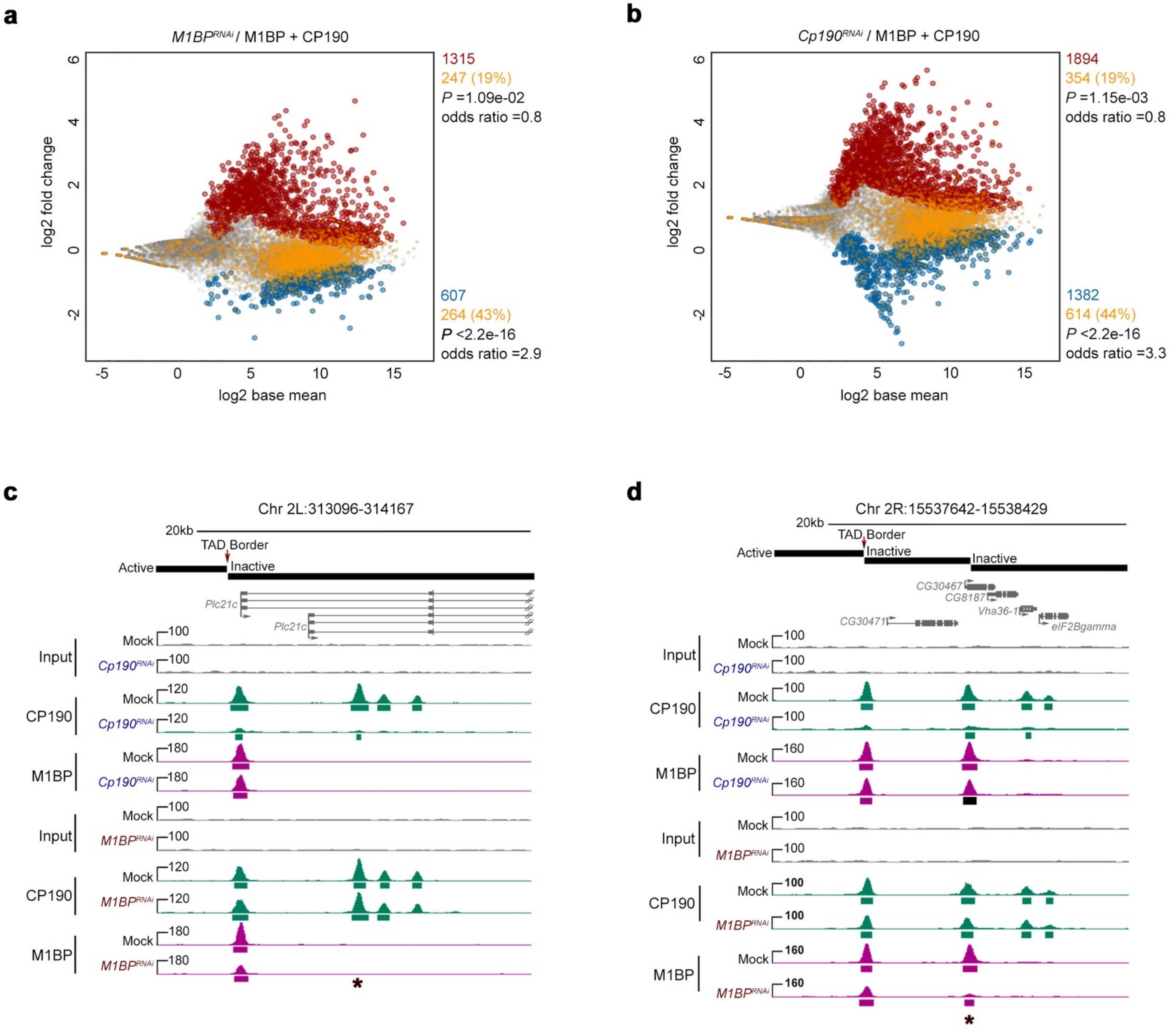

Supplementary Figure 4. Related to Figure 4 and Figure 6. M1BP and CP190 bind promoters of M1BP- and CP190-activated genes and M1BP promotes CP190 recruitment and vice versa.

a MA plot shows changes in neuRNA levels upon depletion of M1BP. Statistically significant changes include 1315 up-regulated genes (red) and 607 down-regulated genes (blue). Gene promoters containing common overlapping peaks of both M1BP and CP190. Two-sided Fisher's exact test used to derive *P*-values and odds ratios indicate significance of enrichment between affected genes and presence of both M1BP and CP190 peaks at promoters. Peak is additionally colored yellow. **b** MA plot representing affected genes in neuRNA seq after depletion of CP910. Significantly up-regulated genes 1894 (red) and 1382 down-regulated genes (blue) are shown. Common M1BP and CP190 peaks associated with promoters of affected genes are colored yellow (Two-sided Fisher's exact test)**.** **c** Example screenshot of ChIP-seq profiles showing there is no change in CP190 binding after depletion of M1BP at a site that is not co-bound by M1BP. Asterisk indicates particular peak measured in Figure 6C, site 12. **d** Example screenshot of ChIP-seq profiles showing there is no change in CP190 binding after depletion of M1BP, and M1BP binding after depletion of CP190 at shared site. Asterisk indicates the particular peak measured in Figure 6C, site 11. C_t values are available in the Source Data 7 for **c-d**. Statistically significant decreased M1BP ChIP-seq peak after depletion of M1BP is shown in black.

Supplementary Figure 5

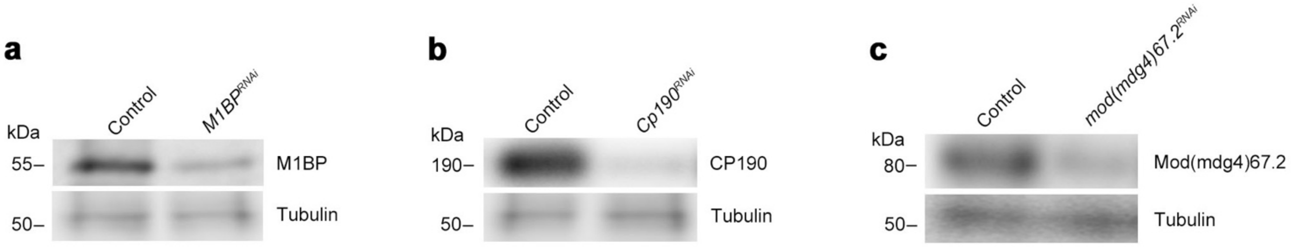

Supplementary Figure 5. Related to Figure 5. dsRNAs are used to deplete M1BP, CP190 and Mod(mdg4)67.2 in Kc cells.

a Western blot showing the knockdown efficiency of *M1BP*,

b *Cp190*,

c *mod(mdg4)*.

Samples from the same experiment were run on different gels for proteins with similar molecular weights. Western blotting was performed two times with similar results.

Supplementary Figure 6

a

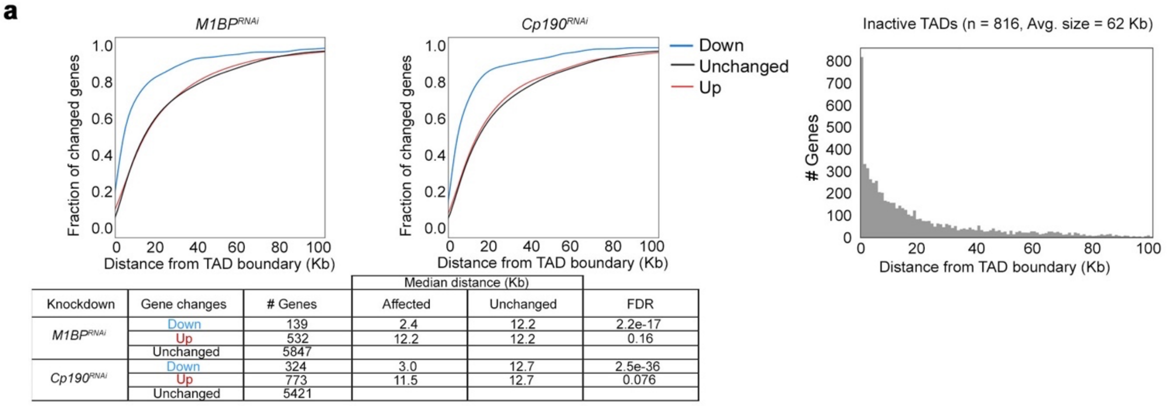

Knockdown	Gene changes	# Genes	Median distance (Kb) Affected	Unchanged	FDR
M1BP^RNAi	Down	139	2.4	12.2	2.2e-17
	Up	532	12.2	12.2	0.16
	Unchanged	5847			
Cp190^RNAi	Down	324	3.0	12.7	2.5e-36
	Up	773	11.5	12.7	0.076
	Unchanged	5421			

b

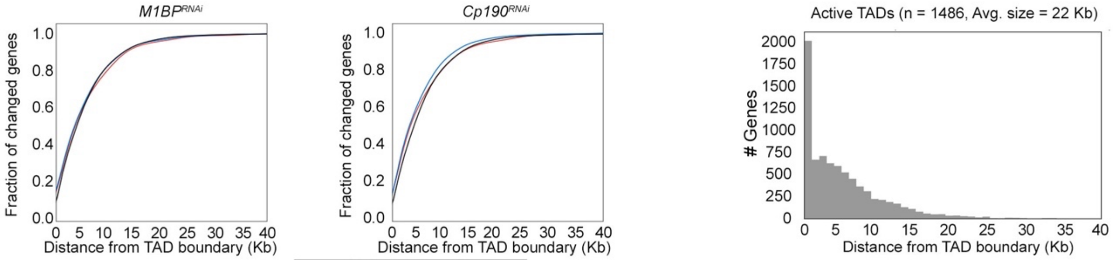

Knockdown	Gene changes	# Genes	Median distance (Kb) Affected	Unchanged	FDR
M1BP^RNAi	Down	417	3.3	3.9	0.020
	Up	462	3.4	3.9	0.11
	Unchanged	6762			
Cp190^RNAi	Down	922	3.0	4.0	5.2e-07
	Up	687	3.0	4.0	3.0e-03
	Unchanged	6032			

c

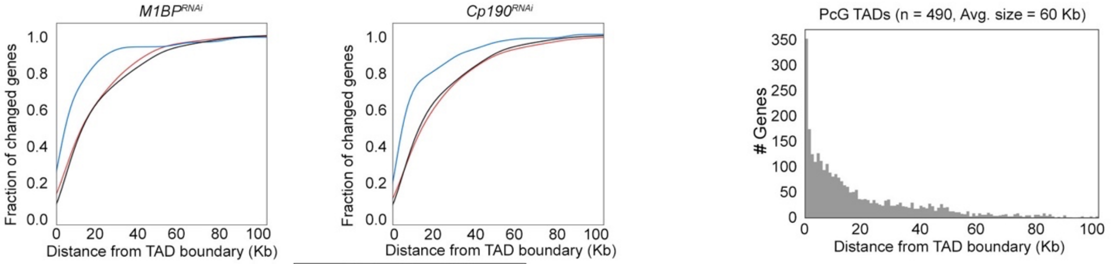

Knockdown	Gene changes	# Genes	Median distance (Kb) Affected	Unchanged	FDR
M1BP^RNAi	Down	40	2.0	11.8	4.3e-06
	Up	265	10.6	11.8	0.058
	Unchanged	2698			
Cp190^RNAi	Down	99	3.3	11.8	5.9e-10
	Up	372	12.7	11.8	0.43
	Unchanged	2532			

d

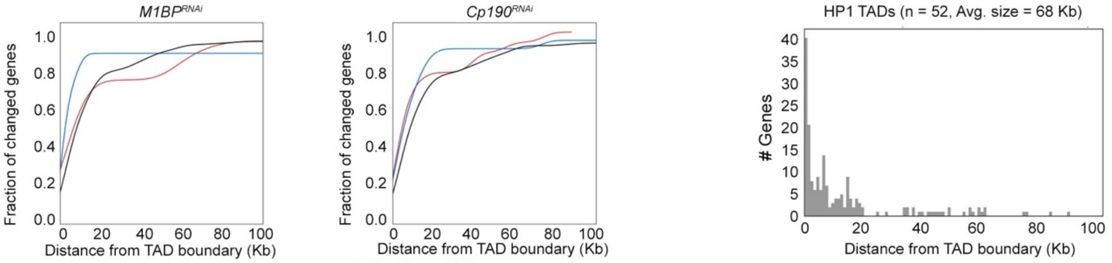

Knockdown	Gene changes	# Genes	Median distance (Kb) Affected	Unchanged	FDR
M1BP^RNAi	Down	9	1.4	6.9	0.057
	Up	20	4.7	6.9	0.18
	Unchanged	168			
Cp190^RNAi	Down	23	2.1	6.9	0.057
	Up	19	1.6	6.9	0.057
	Unchanged	155			

Supplementary Figure 6. Related to Figure 6. M1BP and CP190 transcriptionally activate genes near TAD borders genome-wide independent of chromatin state. Cumulative histograms of promoter distance from closest TAD border for (**a**) inactive, (**b**) active, (**c**) PcG, and (**d**) HP1 TADs classified by change in nascent expression in *M1BP* (left) or *Cp190* (right) knockdown cells. Downregulated (blue), upregulated (red), or unchanged (black) genes are indicated. Tables below each graph indicate number of changed genes for upregulated, downregulated, and unchanged genes for each knockdown and median distance from closest TAD border. Genes with promoters not located inside a TAD were excluded from the analysis. Mann-Whitney U test for each set of changed genes against unchanged genes are shown. Average sizes and number for each TAD class are also shown. Histogram with number of genes per closest distance to each TAD border is shown. Note that active TADs are the shortest and therefore display the least median distance between borders and all gene promoters yet still display statistically significant differences.

Supplementary Figure 7

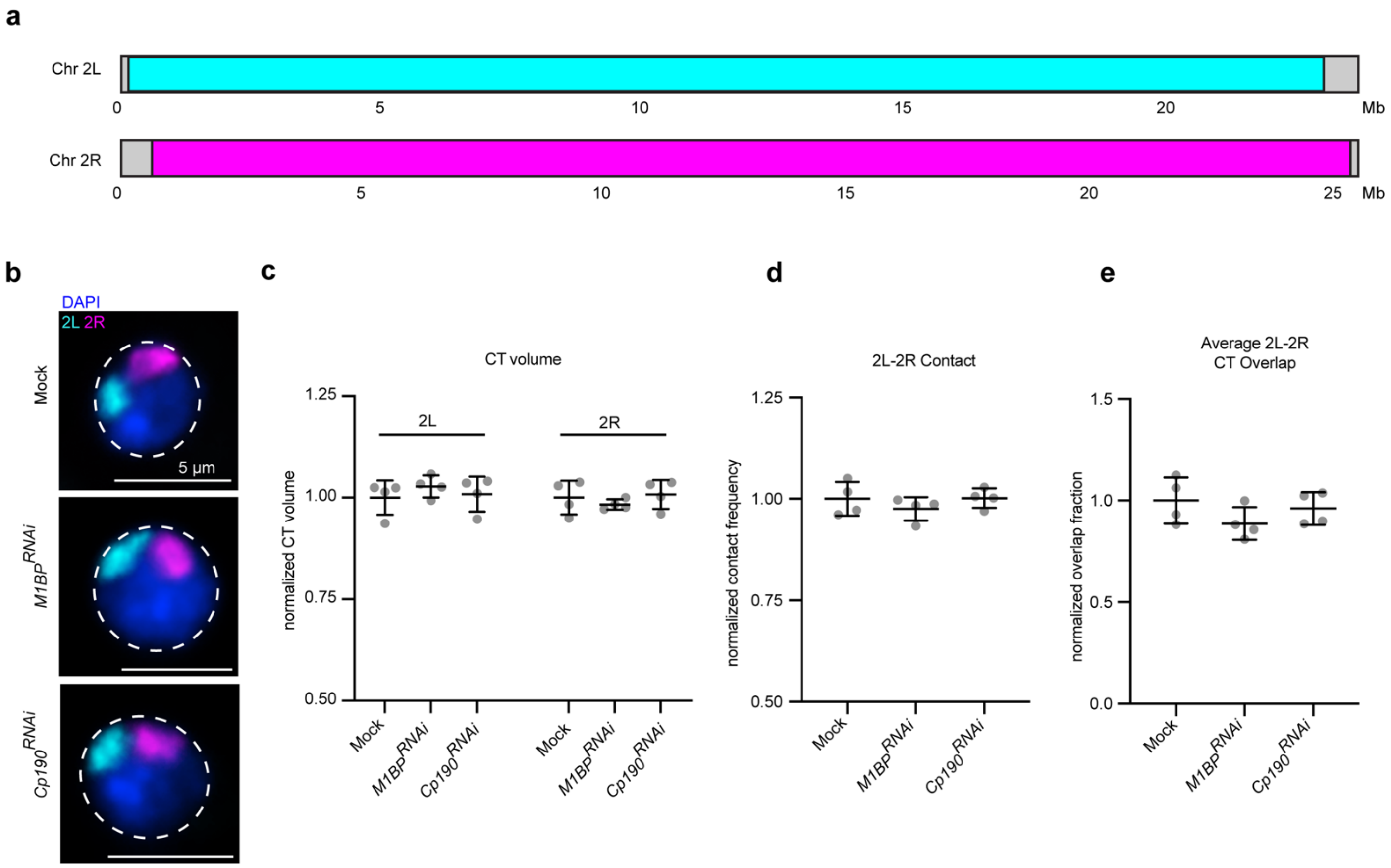

Supplementary Figure 7. Related to Figure 8. Depletion of M1BP or CP190 does not alter CT formation.

a Schematic of whole chromosome paints used in **b-e**. **b** Representative images of CTs in control (Mock) or knockdown cells (*M1BP*, *Cp190*). Images are projections of 10 Z stacks. Dashed line represents nuclear edge. **c** Dot plot showing average CT volume shown as a fraction of nuclear volume normalized to controls. 2L, left; 2R, right. 2L (Mock vs $M1BP^{RNAi}$ P = 0.12, Mock vs $Cp190^{RNAi}$ P = 0.25), 2R (Mock vs $M1BP^{RNAi}$ P = 0.38, Mock vs $Cp190^{RNAi}$ P = 0.25). **d** Absolute contact frequencies between 2L and 2R (where 100% = contact in all cells) normalized to controls. Mock vs $M1BP^{RNAi}$ P = 0.25, Mock vs $Cp190^{RNAi}$ P > 0.99. **e** Average 2L-2R CT intermixing volume as a fraction of 2R volume. All values are normalized to the average of mock controls. Mock vs $M1BP^{RNAi}$ P = 0.25, Mock vs $Cp190^{RNAi}$ P = 0.62. In plots **c-e**, data are represented as mean of all replicates

(mid-line) ± SD (error bars). Each dot represents the mean of a single replicate. P-values determined by paired t-test (two-tailed) of means before normalization to controls for all samples. All data shown in **c-e**, number of cells examined from replicate 1 (Mock n = 726, *M1BP*^{RNAi} n = 406, *Cp190*^{RNAi} n = 508), replicate 2 (Mock n = 575, *M1BP*^{RNAi} n = 336, *Cp190*^{RNAi} n = 656), replicate 3 (Mock n = 368, *M1BP*^{RNAi} n = 641, *Cp190*^{RNAi} n = 608), replicate 4 (Mock n = 845, *M1BP*^{RNAi} n = 843, *Cp190*^{RNAi} n = 863) over four biological replicates.

Supplementary Figure 8

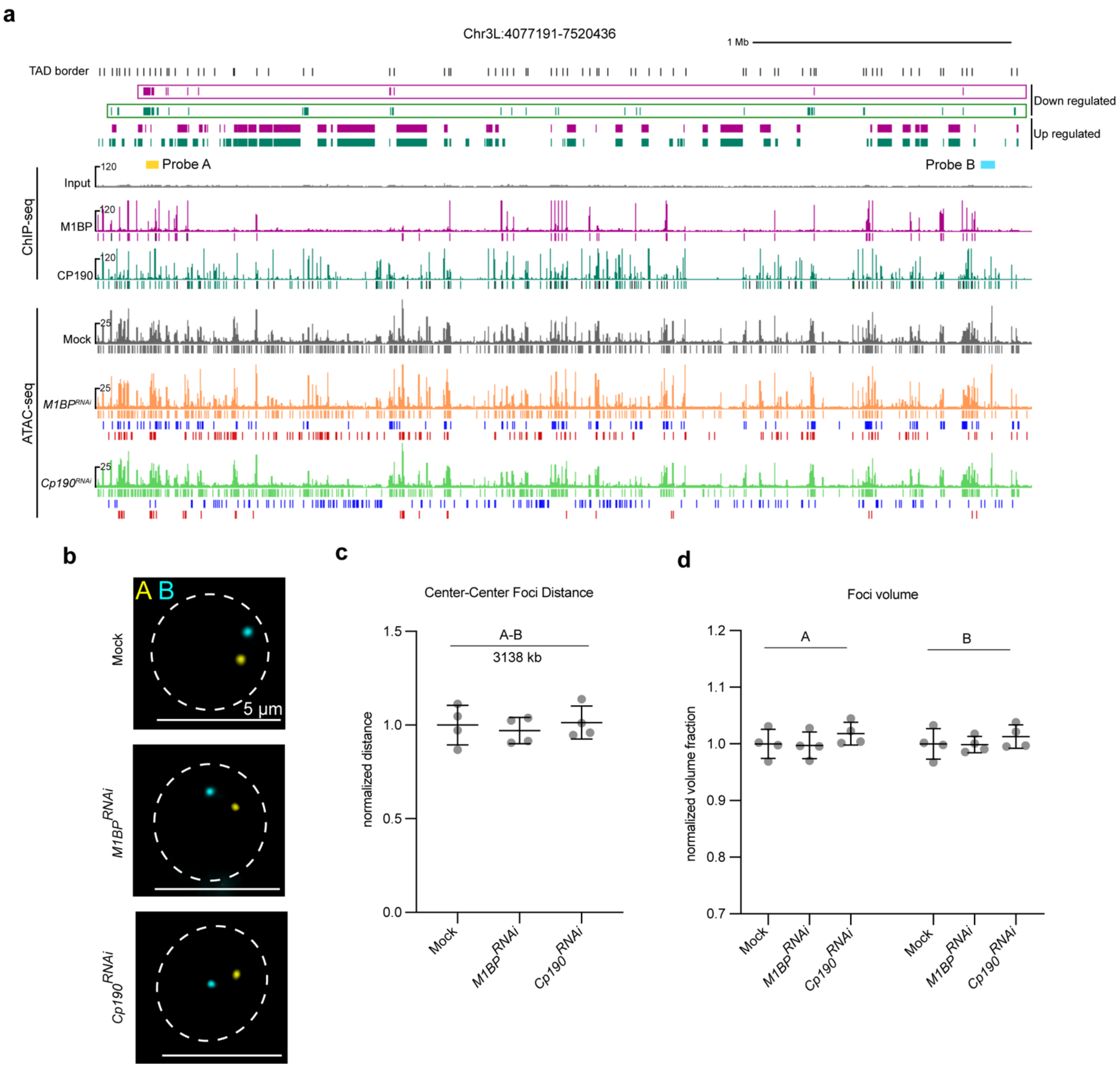

Supplementary Figure 8. Related to Figure 8. Depletion of M1BP or CP190 does not change long-range intra-chromosomal distance.

a Screenshot of regions detected by A and B probes spaced ~3.1 Mb apart. TAD borders, downregulated (boxed) and upregulated genes in either M1BP (purple) or CP190 (green) knockdowns, ChIP-seq signals of M1BP and CP190, and called peaks are shown. Called ChIP-seq peaks shown in black are decreased in the knockdown of the opposite factor. ATAC-seq signals of mock, M1BP-depleted, and CP190-depleted cells, called peaks, decreased peaks relative to mock (blue bars), and increased peaks (red bars) are also shown. **b** Representative nuclei labeled with probes shown in **a**. Single Z slices are shown. Dashed line represents nuclear edge. **c** Average center-to-center distance between probes A and B. Single cell distances were normalized to nuclear radius, and all population averages were normalized to their respective controls. Mock vs $M1BP^{RNAi}$ P = 0.62, Mock vs $Cp190^{RNAi}$ P > 0.99. **d** Normalized foci volume shown as a fraction of nuclear volume. All averages were normalized to the average of mock controls. Probe A (Mock vs $M1BP^{RNAi}$ P = 0.25, Mock vs $Cp190^{RNAi}$ P = 0.25), Probe B (Mock vs $M1BP^{RNAi}$ P > 0.99, Mock vs $Cp190^{RNAi}$ P = 0.62). In plots **c-d**, data are represented as mean of all replicates (mid-line) ± SD (error bars). Each dot represents the mean of a single replicate. P-values determined by paired t-test (two-tailed) of means before normalization to controls for all samples. All data shown in c-d are from four biological replicates. Number of cells measured from replicate 1 (Mock n = 200, $M1BP^{RNAi}$ n = 191, $Cp190^{RNAi}$ n = 191), replicate 2 (Mock n = 192, $M1BP^{RNAi}$ n = 180, $Cp190^{RNAi}$ n = 108), replicate 3 (Mock n = 137, $M1BP^{RNAi}$ n = 196, $Cp190^{RNAi}$ n = 214), replicate 4 (Mock n = 165, $M1BP^{RNAi}$ n = 190, $Cp190^{RNAi}$ n = 191). Data represented as mean ± SD.

Supplementary Figure 9

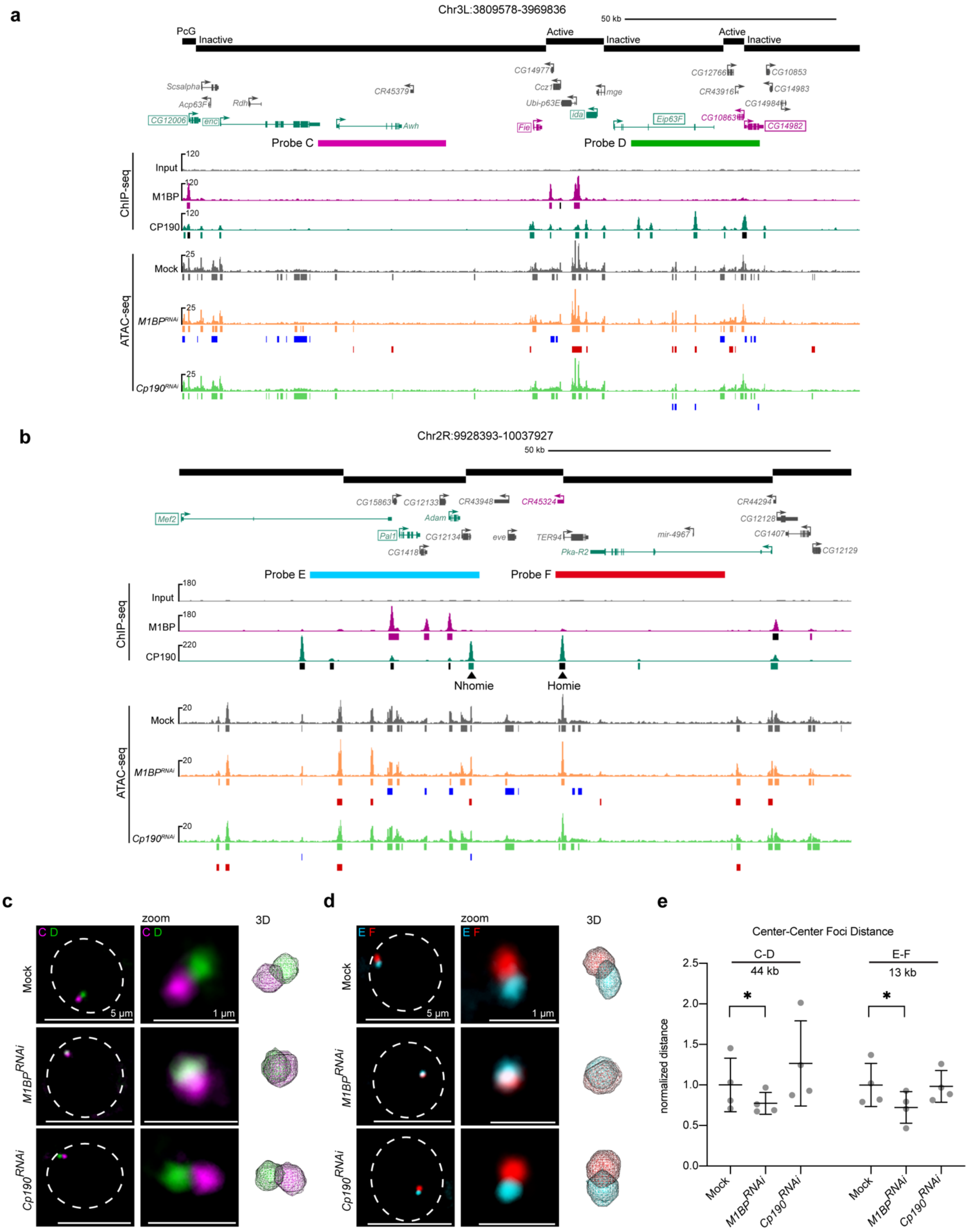

Supplementary Figure 9. Related to Figure 8. Knockdown of *M1BP* increases local genome compaction.

a Screenshot of regions detected by C and D probes spaced ~44 kb apart. TADs with state classification, longest isoform of genes, ChIP-seq signals of M1BP and CP190, and called peaks are shown. Upregulated genes in either *M1BP* or *Cp190* knockdown are shown in purple or green, respectively. Downregulated genes in either *M1BP* or *Cp190* knockdowns are outlined with a box and shown in purple or green, respectively. Called ChIP-seq peaks shown in black are significantly decreased in the knockdown of the opposite factor. ATAC-seq signals of mock, M1BP-depleted, and CP190-depleted cells, called peaks, decreased peaks relative to mock (blue bars), and increased peaks (red bars) are also shown. **b** Screenshot of regions detected by E and F probes spaced ~13 kb apart. **c**, **d** Left: Representative G1 nuclei labeled with probes shown in **a** and **b**, respectively. Single Z slices are shown. Dashed line represents nuclear edge. Center: Zoom of FISH signals. Right: 3D mesh rendering from TANGO. **e** Average center-to-center distance between probes C-D (left) and E-F (right). Single cell distances were normalized to nuclear radius before population averages were calculated. All averages were normalized to the average of mock controls. Asterisk indicates *P*-values < 0.05. C-D (Mock vs $M1BP^{RNAi}$ $P = 0.045$, Mock vs $Cp190^{RNAi}$ $P = 0.11$), E-F (Mock vs $M1BP^{RNAi}$ $P = 0.039$, Mock vs $Cp190^{RNAi}$ $P = 0.97$). Data are represented as mean of all replicates (mid-line) ± SD (error bars). Each dot represents the mean of a single replicate. P-values determined by paired t-test (two-tailed) of means before normalization to controls. Data shown are from G1 cells from four biological replicates, C-D: replicate 1 (Mock n = 144, $M1BP^{RNAi}$ n = 350, $Cp190^{RNAi}$ n = 106), replicate 2 (Mock n = 196, $M1BP^{RNAi}$ n = 239, $Cp190^{RNAi}$ n = 84), replicate 3 (Mock n = 285, $M1BP^{RNAi}$ n = 312, $Cp190^{RNAi}$ n = 206), replicate 4 (Mock n = 187, $M1BP^{RNAi}$ n = 555, $Cp190^{RNAi}$ n = 195), E-F: replicate 1 (Mock n = 92, $M1BP^{RNAi}$ n = 109, $Cp190^{RNAi}$ n = 76), replicate 2 (Mock n = 97, $M1BP^{RNAi}$ n = 161, $Cp190^{RNAi}$ n = 93), replicate 3 (Mock n = 99, $M1BP^{RNAi}$ n = 93, $Cp190^{RNAi}$ n = 127), replicate 4 (Mock n = 190, $M1BP^{RNAi}$ n = 206, $Cp190^{RNAi}$ n = 187). Data represented as mean ± SD.

Supplementary Figure 10

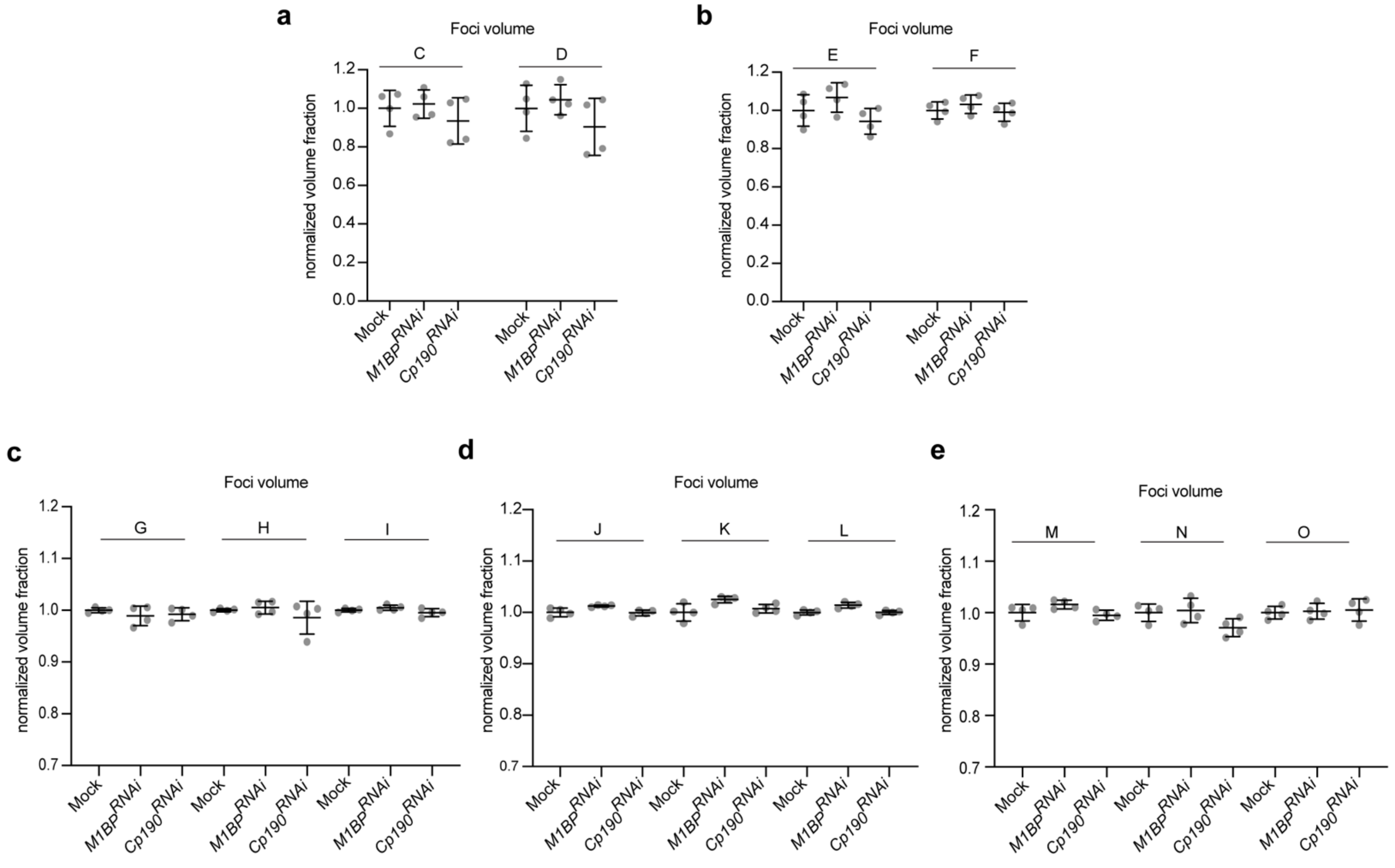

Supplementary Figure 10. Related to Figure 8. Knockdowns of *M1BP* and *Cp190* do not alter foci volume.

a Normalized foci volume shown as a fraction of nuclear volume of Probes C (Mock vs *M1BP*^{RNAi} *P* = 0.72, Mock vs *Cp190*^{RNAi} *P* = 0.42), D (Mock vs *M1BP*^{RNAi} *P* = 0.56, Mock vs *Cp190*^{RNAi} *P* = 0.35), **b** Probes E (Mock vs *M1BP*^{RNAi} *P* = 0.28, Mock vs *Cp190*^{RNAi} *P* = 0.33), F, (Mock vs *M1BP*^{RNAi} *P* = 0.38, Mock vs *Cp190*^{RNAi} *P* = 0.78), **c** Probes G (Mock vs *M1BP*^{RNAi} *P* = 0.34, Mock vs *Cp190*^{RNAi} *P* = 0.32), H (Mock vs *M1BP*^{RNAi} *P* = 0.48, Mock vs *Cp190*^{RNAi} *P* = 0.43), I (Mock vs *M1BP*^{RNAi} *P* = 0.17, Mock vs *Cp190*^{RNAi} *P* = 0.33), **d** Probes J (Mock vs *M1BP*^{RNAi} *P* = 0.060, Mock vs *Cp190*^{RNAi} *P* = 0.88), K (Mock vs *M1BP*^{RNAi} *P* = 0.056, Mock vs *Cp190*^{RNAi} *P* = 0.47), L (Mock vs *M1BP*^{RNAi} *P* = 0.055, Mock vs *Cp190*^{RNAi} *P* = 0.98), **e** M (Mock vs *M1BP*^{RNAi} *P* = 0.99, Mock vs *Cp190*^{RNAi} *P* = 0.96), N (Mock vs *M1BP*^{RNAi} *P* = 0.69, Mock vs *Cp190*^{RNAi} *P* = 0.23) and O (Mock vs

M1BP^{RNAi} *P* = 0.99, Mock vs *Cp190*^{RNAi} *P* = 0.95). In plots **a-e**, data are represented as mean of all replicates (mid-line) ± SD (error bars). Each dot represents the mean of a single replicate. *P*-values determined by unpaired t-test (two-tailed) of means before normalization to controls for all samples. All averages were normalized to the average of their respective mock controls. Data represented as mean ± SD. **a-b**, four biological replicates were used. Please see "n" per replicate in legends of Supplementary Figure 9 and for **c-e** Supplementary Table 10-12.

Supplementary Figure 11

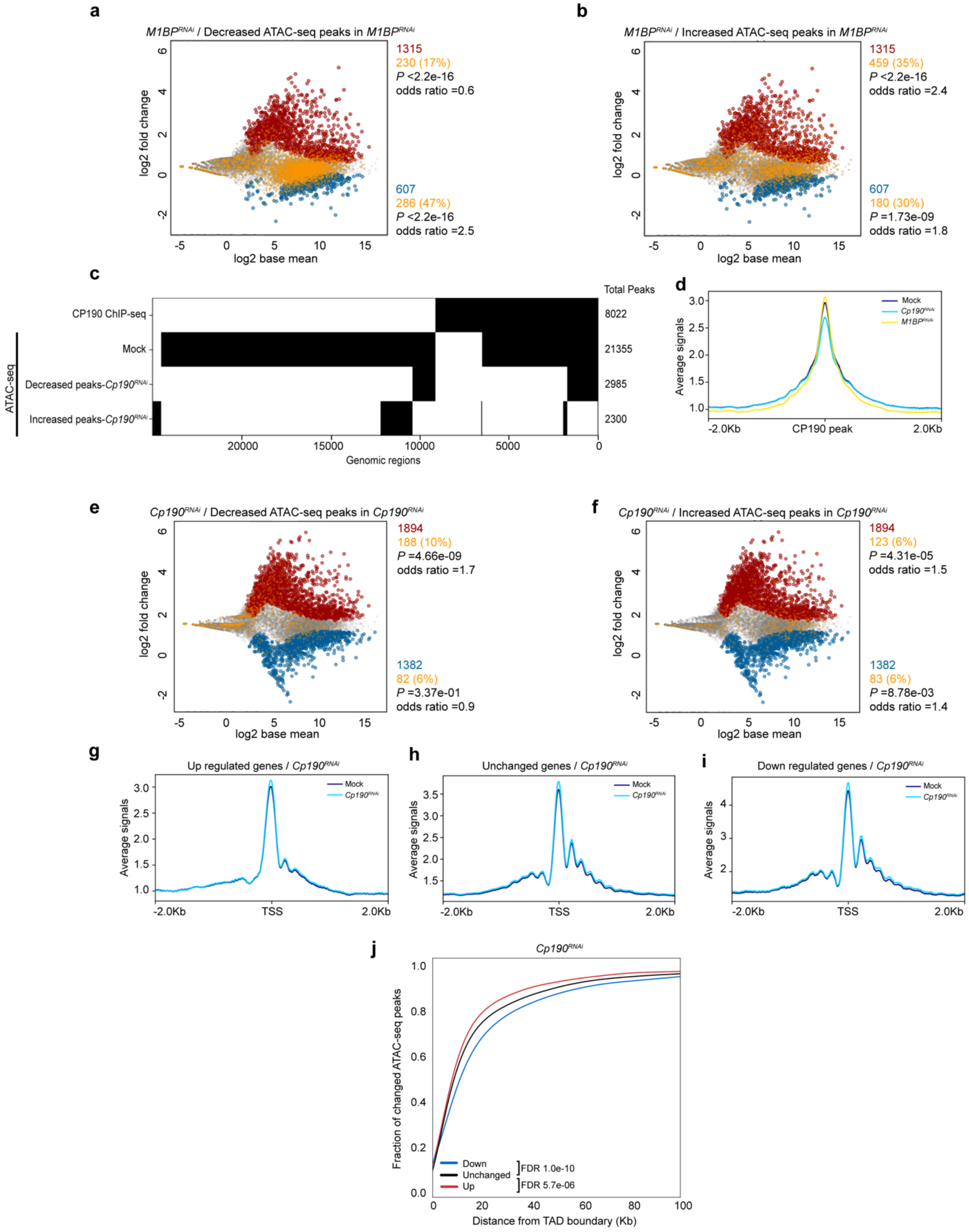

Supplementary Figure 11. Related to Figure 9. *CP190* knockdown shows mild changes in chromatin accessibility genome-wide.

a, **b** MA plots showing changes in neuRNA levels upon depletion of M1BP. Statistically significant changes include 1315 up-regulated genes (red) and 607 down-regulated genes (blue) using $p_{adj} < 0.05$. Unchanged genes are indicated in grey. Fisher's exact test (two-sided) was used. Gene promoters containing decreased ATAC-seq peaks (**a**) or increased ATAC-seq peaks in depletion of M1BP (**b**) are additionally colored yellow. **c** Binary heatmap of CP190 ChIP-seq peaks, Mock ATAC-seq peaks, decreased and increased ATAC-seq peaks in *Cp190* knockdown ordered by supervised hierarchical clustering. Each row represents a single independent genomic location, and a black mark in a column represents the presence of a particular factor. **d** Average ATAC-seq signals of Mock, $M1BP^{RNAi}$ and $Cp190^{RNAi}$ from a 4 kb genomic window centered on CP190 binding sites (ChIP-seq peaks). **e**, **f** MA plots showing changes in neuRNA levels upon depletion of CP190. Statistically significant changes include 1894 up-regulated genes (red) and 1382 down-regulated genes (blue) using $p_{adj} < 0.05$. Unchanged genes are indicated in grey. Fisher's exact test (two-sided) was used. Gene promoters containing decreased ATAC-seq peaks (**e**) or increased ATAC-seq peaks in depletion of CP190 (**f**) are additionally colored yellow. **g**, **h**, **i** Average signal plots show enrichement of ATAC-seq signals of Mock, $M1BP^{RNAi}$ and $Cp190^{RNAi}$ from a 4 Kb genomic window at TSS of upregulated genes (**g**) Unchanged genes (**h**) Downregulated genes (**i**) in *Cp190* knockdown**. j** Cumulative histograms of $Cp190^{RNAi}$-ATAC-seq peak center distance from closest TAD border classified by change in nascent expression in *Cp190* knockdown cells. Downregulated (blue), upregulated (red), or unchanged (black) genes are indicated. Mann-Whitney U test for each set of changed genes against unchanged genes are shown.

Supplementary Table 1. Mass-spec with antibody against Su(Hw) using *Drosophila* embryonic nuclear extract. Presence of M1BP is highlighted in yellow.

Ratio H/L Su(Hw)	Score	Intensity	Razor + unique peptides Su(Hw)	Protein IDs	Protein names	Gene names
212.04	323.31	8594600000	19	A0A0B4K6W1;P08970	Protein suppressor of hairy wing	su(Hw)
166.26	323.31	1660900000	17	Q95RQ8;Q9W1N1		pita
95.362	63.599	84784000	4	Q9V3G3	Peptidyl-prolyl cis-trans isomerase E	cyp33
92.033	323.31	1.6513E+10	35	Q24478	Centrosome-associated zinc finger protein CP190	Cp190
87.248	13.871	789000000	2	Q9V9X7		CG11337-RD
71.067	323.31	1295600000	9	A0A0B4LGV6		Ibf1
70.513	98.816	665430000	4	Q9VHG6		Ibf2
68.41	103.67	36319000	3	A0A0B4K7R5;A0A0B4K807;Q9NKV0-2;Q9NKV0-4;Q9NKV0;Q9NKV0-3	Myeloid leukemia factor	Mlf
65.765	6.1137	7251700000	1	A1Z6H9		dpr12
60.171	323.31	4327600000	16	Q86B87;Q86B87-22;Q86B87-26;A0A0B4JDE0	Modifier of mdg4	mod(mdg4)
52.474	47.796	33434000	3	P38040	Guanine nucleotide-binding protein subunit gamma-1	Ggamma1
51.558	19.674	370870000	3	X2JIQ5;Q9W3W8	60S ribosomal protein L17	RpL17
49.827	57.753	21065000	4	Q9VRQ7	DNA polymerase epsilon subunit 2	DNApol-epsilon58
47.735	140.93	851980000	5	Q94513		BEAF-32
45	39.496	191410000	5	P07664	Serendipity locus protein delta	Sry-delta
43.222	67.193	73308000	5	Q9V3Z3		meso18E
42.194	323.31	1386900000	17	Q6NP69;Q8INS9		gfzf
42.132	323.31	567010000	11	Q9VHM3		M1BP
41.036	323.31	89727000	8	Q8IMF8;A0A0B4LHY1;Q8WR53;Q7KRS7;A8JRI1;Q95UI6;A0A0B4K6P4;A0A0B4K6W9;Q7KES3;E1JJ45	heph	
41.03	118.36	173600000	7	Q9VS55		CTCF
39.906	237.2	135230000	7	Q9V3D6	Probable cleavage and polyadenylation specificity factor subunit 2	Cpsf100
38.81	246.39	84490000	8	Q9VDY1	Putative DNA helicase Ino80	Ino80
37.27	47.598	64998000	4	E0R905;Q7KSP5;A0A0B4KGA3;A0A0B4K6S1;A0A0B4K6F4;Q8SZN9	l(3)neo38-RA;l(3)neo38	
37.193	75.745	65045000	5	Q9VQ58		CG15356-RA
35.987	46.804	21145000	3	M9PBX0;P42003;O96660	Mothers against decapentaplegic homolog;Protein mothers against dpp	Mad
34.63	210.89	46421000	4	Q9VPX5	Vacuolar protein sorting-associated protein 29	Vps29
34.145	20.806	16203000	3	Q9W268;Q8MLT6		dve
34.117	323.31	1119500000	28	Q9VZ00;X2JEU3		CG1737-RA
32.819	323.31	320460000	16	E1JH59;A1Z9E2;A1Z9E2-2	Protein lin-54 homolog	mip120
32.745	323.31	248720000	17	Q961B8;A0A0B4LEU5	ADD1	
32.497	323.31	102770000	7	Q9VVA6		nudC
32.053	323.31	39317000	5	B7Z0G1;Q02870	DNA excision repair protein haywire	hay
31.819	19.338	95692000	3	O76857		BCL7-like

31.502	147.01	382590000	11	O61602;Q9W1M6;A0A0B4KGD0;Q8MLR4	apt	
31.338	25.987	378770000	2	O44437	Small nuclear ribonucleoprotein Sm D3	SmD3
31.258	16.62	31396000	2	Q8MRW1		Tim23
31.225	94.207	142350000	3	Q9VFB5		Rpb7
31.214	31.916	25071000	2	Q9VQG4;Q9VM51	Congested-like trachea protein	colt
30.873	74.034	60741000	5	Q9VN50	Eukaryotic translation initiation factor 3 subunit F-1	eIF3-S5-1
30.367	24.182	70674000	3	P22769	Proteasome subunit alpha type-7-1	Prosalpha4
30.276	33.388	27072000	3	Q9W1V8		l(2)06496
30.095	66.955	80908000	8	B7YZI0;Q9V7N5-2;Q9V7N5-3;Q9V7N5	V-type proton ATPase subunit C	Vha44
30.026	110.2	53398000	5	Q9VTU0	Protein KRI1 homolog	CG5645
29.971	323.31	2690000000	56	M9PE12;E1JHV6;M9PDH1;Q9W0T1;Q9W0T1-2;M9PBG5;Q9W0T1-3;E1JHV7;Q9W0T1-4	Nucleosome-remodeling factor subunit NURF301	E(bx)
29.815	103.89	34450000	5	X2JA36;Q9VKM6	Vacuolar protein sorting-associated protein 72 homolog	YL-1
29.692	39.542	24197000	2	Q9VB49		mrt
29.672	188.86	24996000	3	Q9N693;E1JGL0;A0A0B4KEU2;Q9NBW0;A1ZB51	sbb	
29.313	27.346	17808000	3	Q7JUX9	Mitochondrial ribonuclease P protein 1 homolog	trmt10c
29.253	72.181	1446000000	4	Q9W0A8		RpL23A
29.093	81.43	20018000	4	Q9VGI8	Bloom syndrome protein homolog	Blm

Supplementary Table 2. Mass-spec with antibody against CP190 using *Drosophila* embryonic nuclear extract. Presence of M1BP is highlighted in yellow.

Ratio H/L CP190	Score	Intensity	Razor + unique peptides CP190	Protein IDs	Protein names	Gene names
1370.2	240.72	4038100000	12	Q9VS55		CTCF
523.26	173.99	1119300000	30	M9MS40;Q9W321; M9PH39		nej
523.24	225.63	7975900000	7	Q94513		BEAF-32
477.82	87.812	338600000	3	Q86B87-12	Modifier of mdg4	mod(mdg4)
358.8	6.315	23477000	1	Q7JWH5		Roc2
342.74	46.197	2677100000	8	P07664	Serendipity locus protein delta	Sry-delta
321.29	244.59	8741700000	14	Q9VHM3		M1BP
298.11	5.6454	2003800000	2	P48588	40S ribosomal protein S25	RpS25
254.74	155.88	826530000	19	Q9V3P2		l(2)34Fd
237.09	122.93	428110000	17	Q9VKJ1	MPN domain-containing protein CG4751	CG4751
229.62	276.24	437250000	12	Q9VF00;A0A0B4JDD2		GATAe
225.25	282.5	592970000	11	Q7K2K2;A0A0C4DHG4		CG10265-RB
223.97	12.179	1270600000	5	M9PCI2;Q9V3U2	H/ACA ribonucleoprotein complex subunit 2-like protein	NHP2
216.59	323.31	75932000000	26	Q9VP57		pzg
213.05	323.31	2.36E+11	41	Q24478	Centrosome-associated zinc finger protein CP190	Cp190
213.01	150.85	172400000	3	Q9NDJ2-2	Helicase domino	dom
201.73	323.31	24760000000	20	A0A0B4K6W1;P08970	Protein suppressor of hairy wing	su(Hw)
197.23	323.31	14583000000	65	M9PDH1;M9PE12;E1JHV6; Q9W0T1;Q9W0T1-2;M9PBG5	Nucleosome-remodeling factor subunit NURF301	E(bx)
196.61	91.73	835900000	25	Q9VUQ5-2;Q9VUQ5;M9PFK7	Protein argonaute-2	AGO2
173.41	45.172	1371100000	6	Q867Z4	Longitudinals lacking protein, isoforms F/I/K/T	lola
170.35	323.31	20926000000	24	Q86BS3		Chro
170.3	323.31	3389900000	24	M9PID3;M9PG82;M9PG20; Q9VPB0		HIPP1
167.67	81.458	7974200000	7	Q9VHG6		Ibf2
164.1	71.81	151150000	6	Q7K148	Proteasome subunit beta type	Prosbeta5
146.68	323.31	10000000000	26	Q6NP69;Q8INS9		gfzf
145.58	13.771	46507000	7	Q9VBA2		SIP2-RE
145.13	26.461	334110000	3	X2JKS9;Q9VXB3	Histone deacetylase complex subunit SAP30 homolog	Sap30
143.66	20.997	187390000	10	Q9VR99	Cactin	cactin
135.29	121.04	827160000	6	E0R905;Q7KSP5;A0A0B4KGA3		l(3)neo38-RA;l(3)neo38
130.24	91.258	510650000	9	Q9V9N4;Q8IGP5		Jupiter;Clamp
126.26	19.669	244850000	7	Q9XYA7;Q8INR0;Q8INQ9		stck
123.78	251.21	15211000000	10	A0A0B4LGV6		Ibf1
122.29	323.31	2504300000	30	E1JH59;A1Z9E2;A1Z9E2-2	Protein lin-54 homolog	mip120
113	108.33	215630000	5	Q9W4V9		dwg
111.91	45.803	52667000	4	Q9VFX8		Ravus
110.91	61.461	218180000	10	M9PCX8;M9PCD8;P08630-2;P08630	Non-specific protein-tyrosine kinase;Tyrosine-protein kinase Btk29A	Btk29A
109.48	63.299	175110000	16	X2JEY9;M9PHF1;X2JEM2; X2JJP5;A8JUU1;A8JUU3		Sec16

105.36	89.551	420650000	11	Q7K4M4	Protein teflon	tef
103.69	200.11	1192700000	17	Q9VRI0		Hlc
102.54	108.68	267410000	9	Q9VDS7;A0A0B4LID7		CG4538-RA
99.278	26.326	54268000	7	Q24371-2;Q24371	Protein lethal(2)denticleless	l(2)dtl
97.531	270.1	1188400000	13	Q9VZJ3		Rcd5
90.255	50.377	117030000	5	Q9W2E3		NC2alpha
89.241	52.522	216100000	8	Q0KI58;A0A0B4KHP7;Q8IN90;Q5BIJ2;B7Z0L6;A0A0B4KHB9;B7Z0L7;Q9VEC6;Q8IN89		PP2A-B
88.634	43.088	1053500000	4	Q9VN42		Dip2
86.12	22.681	1592400000	2	Q9VBH8;Q9VHE5		RpL34a;RpL34b
84.735	323.31	5389700000	31	Q9VXE9	Structural maintenance of chromosomes protein	SMC3
82.524	5.3231	88782000	2	Q9XY35	Cytochrome b-c1 complex subunit 9	ox
79.162	20.843	205930000	5	Q9W0S6		mRpL17
77.842	9.1999	115120000	5	Q9VA37-2;Q9VA37		dj-1beta

Supplementary Table 3. All viability or lethality levels were 100% unless otherwise indicated. n=total number of flies tested.

Knockdown	*Act5C-Gal4* (ubiquitous)	*Mef2-Gal4* (muscle-specific)	*l(3)31-Gal4* (CNS-enriched)	*Ser-Gal4* (various tissues)
$M1BP^{RNAi}$	Late larval lethality (n=150)	Pupal lethality (n=100)	Viable (n=100)	Viable (n=120)

Supplementary Table 4. Luciferase assay *P*-values for *M1BP* knockdown and all other controls using *Act5C-Gal4*, *Mef2-Gal4* and *l(3)31-1-Gal4* in male larvae are indicated. *Gal4* driver lines are highlighted with orange. All genotypes of larvae are indicated with bold and italic.

Act5C-Gal4>						
	non-ins; ctrl	***non-ins; su(Hw)***	***non-ins; M1BP^{RNAi}***	***ins; ctrl***	***ins; su(Hw)***	***ins; M1BP^{RNAi}***
non-ins; su(Hw)	9.62E-01					
non-ins; M1BP^{RNAi}	9.74E-01	9.99E-01				
ins; ctrl	1.46E-12	1.46E-12	1.46E-12		1.46E-12	4.43E-05
ins; su(Hw)	1.55-E-02	1.12E-01	9.48E-02	1.46E-12		1.60E-12
ins; M1BP^{RNAi}	1.46E-12	1.46E-12	1.46E-12	4.43E-05	1.60E-12	
Mef2-Gal4>						
	non-ins; ctrl	***non-ins; su(Hw)***	***non-ins; M1BP^{RNAi}***	***ins; ctrl***	***ins; su(Hw)***	***ins; M1BP^{RNAi}***
non-ins; su(Hw)	8.99E-01					
non-ins; M1BP^{RNAi}	6.50E-01	9.97E-01				
ins; ctrl	1.46E-12	1.46E-12	1.46E-12		1.46E-12	2.71E-09
ins; su(Hw)	1.00E+00	9.01E-01	6.48E-01	1.46E-12		1.50E-12
ins; M1BP^{RNAi}	1.51E-12	1.47E-12	1.46E-12	2.71E-09	1.50E-12	
l(3)31-1-Gal4>						
	non-ins; ctrl	***non-ins; su(Hw)***	***non-ins; M1BP^{RNAi}***	***ins; ctrl***	***ins; su(Hw)***	***ins; M1BP^{RNAi}***
non-ins; su(Hw)	1.11E-03					
non-ins; M1BP^{RNAi}	2.58E-01	3.20E-01				
ins; ctrl	1.46E-12	1.19E-11	1.49E-12		1.46E-12	1.56E-02
ins; su(Hw)	9.87E-01	7.70E-05	5.55E-02	1.46E-12		1.52E-12
ins; M1BP^{RNAi}	2.08E-12	8.14E-06	2.10E-02	1.56E-02	1.52E-12	

Supplementary Table 5. Differential binding of CP190 in *M1BP* knockdown and differential binding of M1BP in *Cp190* knockdown are verified by directed ChIP-qPCR and differential binding of Su(Hw), CP190, Mod(mdg4)67.2 in *M1BP* knockdown and in *Cp190* knockdown are tested by ChIP-qPCR.

				M1BP^{RNAi}	*M1BP^{RNAi}*	*Cp190^{RNAi}*	*Cp190^{RNAi}*		
Site name	Chromosome site	M1BP peaks	CP190 peaks	M1BP decreased/ lost in *M1BP^{RNAi}*	CP190 decreased/ lost in *M1BP^{RNAi}*	M1BP decreased/lost in *Cp190^{RNAi}*	CP190 decreased/lost in *Cp190^{RNAi}*	Forward primer	Reverse primer
1	chr2L:285520-286009	Yes	Yes	Yes	Yes	Yes	Yes	GGGGAACTGCACGTAGTTGT	GGTGCCGTGAAAAATCAATC
2	chr2L:4955229-4955768	Yes	Yes	Yes	Yes	Yes	Yes	TCCGAGATAGCGGAGAAGAA	CGTCGGTGATCGGTAGAAAT
3	chr3L:9360165-9360542	Yes	Yes	Yes	Yes	Yes	Yes	CATGGGGGTGGTGATAAAAG	GCGGAGATTACCGAATTGAA
4	chr2L:8082778-8083790	Yes	Yes	Yes	Yes	Yes	Yes	CTCGGGAACTCAAAGATTCG	GTTCTGGCCACACCTGAAAT
5	chrX:21339252-21340005	Yes	Yes	Yes	Yes	Yes	Yes	ACCGTTAAAAGCCATGCAAA	TGGAAATCACAGAAGGCACA
6	chr2R:19304896 -19305345	Yes	Yes	Yes	Yes	No	Yes	TACTCCGGTACTCGGTGCTC	TGTGCCTCCACATAGTTTCG
7	chr2R:20976497-20977464	Yes	Yes	Yes	Yes	No	Yes	GGCACACGATCCAAAGTACC	TCACTTGCCTTTTCCTTGCT
8	chr2L:285520-286009	Yes	Yes	Yes	Yes	No	Yes	GGGGAACTGCACGTAGTTGT	GTTTTGGTGCCGTGAAAAAT
9	chr3R:31745692-31746393	Yes	Yes	Yes	Yes	No	Yes	TCAGGAGCTTCATGTGTTCG	CTCAAATTTCCGCTTTGGAA
10	chrX:17702652-17703572	Yes	Yes	Yes	No	No	Yes	CGCTACAATTGGGGCATATT	TTCAGATGCGCTACATTTGC
11	chr2R:15537642-15538429	Yes	Yes	Yes	No	No	Yes	ACCGAAGTGCGAGAAAGTGT	CAGAATGCAGAATGGCAGTG
12	chr2L:313096-314167	No	Yes	No	No	No	Yes	AAATTACCAGCCGAATGCAC	GCCATAAATCGTCTGCCACT
gypsy	N/A	N/A	N/A	N/A	N/A	N/A	N/A	TCAAAAAATAAGTGCTGCATACTTTTTAG	AGCACAATTGATCGGCTA
TART	N/A	N/A	N/A	N/A	N/A	N/A	N/A	GTACAACCAAAGTTGACGGG	TGTATCGATATTTCGCGCTTTT
Rint1	chr3L:7322619-7324619	Yes	Yes	Yes	Yes	No	Yes	TTGTGTGCTGCTCACCTTTC	GTGTGGCGTCGTTTTCTTTT
CG7872	chrX:15577205-15579205	Yes	Yes	Yes	Yes	No	Yes	AATCGGTATTTGGCGAACTG	AACACCGATAACCGCGATAG
CG6454	chr3R:24348182-24350182	Yes	Yes	Yes	Yes	No	Yes	GTTGTCATCCTTCAGCGACA	ACTCATTCCCTCCGGAGTTT
tapas	chr2R:20318180-20320180	Yes	Yes	Yes	Yes	No	Yes	GCCCACGGTCTCACTAGAAG	TGGGGTTTTCCTGAGAAATG
PDZ-GEF	chr2L:6322767-6324767	Yes	Yes	Yes	Yes	Yes	Yes	TAACCGATTCCCAGATCAGC	AGTCCGACGCGTCTTACAGT

Supplementary Table 6. List of primers for generation of dsRNA against *M1BP*, *Cp190* and *mod(mdg4).*

Knockdown	Forward primer	Reverse Primer
M1BP^{RNAi}	TAATACGACTCACTATAGGGAAGTTA TTCGAGCGGAGCAA	TAATACGACTCACTATAGGGTAG ACGTACGCGTCGTTGAC
Cp190^{RNAi}	TAATACGACTCACTATAGGGAGA GTAAACGGACGACCCATTAGCATTC	TAATACGACTCACTATAGGGAGA GTCTGCTCTGGTTCCTGCTC
mod(mdg4)67.2^{RNAi}	TAATACGACTCACTATAGGGAGA AACAAAGGTTGAGGATCAGAC	TAATACGACTCACTATAGGGAGA TGGGGTCCACTTCTTCCTC

Supplementary Table 7. List of antibodies used for western blots, immunofluorescence and chromatin immunoprecipitation.

Antibody	Dilution for WB	Dilution for IF	Dilution for ChIP	Resources
Guinea pig anti-CP190	1:10000	—	—	Matzat et al., 2012, Elissa Lei Lab, NIDDK, NIH
Rabbit anti-CP190	—	1:5000	1:333	Moshkovich et al., 2011, Elissa Lei Lab, NIDDK, NIH
Guinea pig anti-Su(Hw)	1:5000	—	1:333	Moshkovich and Lei, 2010, Elissa Lei Lab, NIDDK, NIH
Rabbit anti-Mod(mdg4)67.2	1:1000	—	1:333	Van Bortle et al., 2014, Elissa Lei Lab, NIDDK, NIH
Rabbit anti-M1BP	1:1000	—	1:200	Li and Gilmour, 2013, David Gilmour Lab, Pennsylvania State University
Rabbit anti-Pc	1:8000	—	—	Moshkovich et al., 2011, Patrick H. O'Farrell Lab, UCSF
Mouse anti-Pep	1:1000	—	—	Amero et al., 1991, Ann L. Beyer Lab, University of Virginia School of Medicine
Rabbit anti-BEAF-32	1:1000	—	—	Bushey et al., 2009, Victor Corces Lab, Emory University
Rabbit anti-Histone H3(phospho S10)	—	1:5000	—	Abcam Cat No ab5176
Mouse anti-Tubulin		1:500	—	Sigma Cat No T6074

Supplementary Table 8. List of probes used for FISH experiments.

Probe	Chromosome	Start	Stop	Domain Size (bp)
A	Chr3L	4204273	4254767	50494
B	Chr3L	7393235	7447744	54509
C	Chr3L	3841773	3872172	30399
D	Chr3L	3915948	3946359	30411
E	Chr2R	9944392	9974392	30000
F	Chr2R	9987751	10017741	29990
G	Chr3L	18906473	18938719	32246
H	Chr3L	18953719	18985965	32246
I	Chr3L	19000965	19033211	32246
J	Chr3R	13402320	13435028	32708
K	Chr3R	13450028	13482736	32708
L	Chr3R	13497736	13530444	32708
M	Chr3R	29636892	29669392	32500
N	Chr3R	29684392	29716892	32500
O	Chr3R	29731892	29764392	32500

Supplementary Table 9. List of primers used for probe synthesis.

Probe	Forward (F) Primer Sequence	Reverse (R) Primer Sequence
A	CACCGACGTCGCATAGAACGGAAGAGC GTGTGACAGATCGACGACGGGTTTG	TAATACGACTCACTATAGGGTCCGGGTTAA TCGGTTCGAC
B	TAGCGCAGGAGGTCCACGACGTGCAAG GGTGTCGGAAGCGCGGTTATTGTGC	TAATACGACTCACTATAGGGTCCGGGTTAA TCGGTTCGAC
C	GGTGTGGCTCGGTATCGTGCAAGGGTG AATGCCCATTGCGGCCGGTTGTTAC	TAATACGACTCACTATAGGGTCCGGGTTAA TCGGTTCGAC
D	TAGCGCAGGAGGTCCACGACGTGCAAG GGTGTAACGCCGGTCTCCCGTTATC	TAATACGACTCACTATAGGGTCCGGGTTAA TCGGTTCGAC
E	CACCGACGTCGCATAGAACGGAAGAGC GTGTGTTGACGTTTGCGCCGAATAC	TAATACGACTCACTATAGGGCCGGCCGCA GGTTATATTCC
F	GGTGTGGCTCGGTATCGTGCAAGGGTG AATGCATACAAACCGGGCGGTTCAC	TAATACGACTCACTATAGGGCCGGCCGCA GGTTATATTCC
G	GGTGTGGCTCGGTATCGTGCAAGGGTG AATGCGTCGTCTTGACGCGCATGTG	TAATACGACTCACTATAGGGCACGGCGGA GGGATAAGTTG
H	TAGCGCAGGAGGTCCACGACGTGCAAG GGTGTCCTCGGCCTTTGCGACTAAC	TAATACGACTCACTATAGGGCACGGCGGA GGGATAAGTTG
I	CACACGCTCTCCGTCTTGGCCGTGGTC GATCAGGTAATTCGCCGCCCATAGG	TAATACGACTCACTATAGGGCACGGCGGA GGGATAAGTTG
J	GGTGTGGCTCGGTATCGTGCAAGGGTG AATGCCCGGCCGCAGGTTATATTCC	TAATACGACTCACTATAGGGCACGGCGGA GGGATAAGTTG
K	TAGCGCAGGAGGTCCACGACGTGCAAG GGTGTCATATCGGCGCGGGATGTAC	TAATACGACTCACTATAGGGCACGGCGGA GGGATAAGTTG
L	CACACGCTCTCCGTCTTGGCCGTGGTC GATCACTGCGCGGCCCGAGTTATTG	TAATACGACTCACTATAGGGCACGGCGGA GGGATAAGTTG
M	GGTGTGGCTCGGTATCGTGCAAGGGTG AATGCCTGATGCACCCGCGAGATTC	TAATACGACTCACTATAGGGCACGGCGGA GGGATAAGTTG
N	TAGCGCAGGAGGTCCACGACGTGCAAG GGTGTGTGTCGCGTCGGCCAGAAAC	TAATACGACTCACTATAGGGCACGGCGGA GGGATAAGTTG
O	CACACGCTCTCCGTCTTGGCCGTGGTC GATCACCCGATACGTCGTGGGATTC	TAATACGACTCACTATAGGGCACGGCGGA GGGATAAGTTG

Supplemental Table 10. Number of cells (n) measured per replicate in Mock, *M1BP*^{RNAi}, and *Cp190*^{RNAi} treated cells for probes G, H, I.

Probes	Replicate	Knockdown	"n"
G, H, I	1	Mock	397
G, H, I	2	Mock	353
G, H, I	3	Mock	211
G, H, I	4	Mock	160
G, H, I	1	*M1BP*^{RNAi}	344
G, H, I	2	*M1BP*^{RNAi}	369
G, H, I	3	*M1BP*^{RNAi}	368
G, H, I	4	*M1BP*^{RNAi}	184
G, H, I	1	*Cp190*^{RNAi}	521
G, H, I	2	*Cp190*^{RNAi}	694
G, H, I	3	*Cp190*^{RNAi}	464
G, H, I	4	*Cp190*^{RNAi}	355

Supplemental Table 11. Number of cells (n) measured per replicate in Mock, *M1BP*^{RNAi}, and *Cp190*^{RNAi} treated cells for probes J, K, L.

Probes	Replicate	Knockdown	"n"
J, K, L	1	Mock	279
J, K, L	2	Mock	285
J, K, L	3	Mock	169
J, K, L	4	Mock	172
J, K, L	1	*M1BP*^{RNAi}	235
J, K, L	2	*M1BP*^{RNAi}	237
J, K, L	3	*M1BP*^{RNAi}	105
J, K, L	4	*M1BP*^{RNAi}	174
J, K, L	1	*Cp190*^{RNAi}	453
J, K, L	2	*Cp190*^{RNAi}	269
J, K, L	3	*Cp190*^{RNAi}	141
J, K, L	4	*Cp190*^{RNAi}	269

Supplemental Table 12. Number of cells (n) measured per replicate in Mock, *M1BP*^{RNAi}, and *Cp190*^{RNAi} treated cells for probes M, N, O.

Probes	Replicate	Knockdown	"n"
M, N, O	1	Mock	488
M, N, O	2	Mock	259
M, N, O	3	Mock	672
M, N, O	4	Mock	216
M, N, O	1	*M1BP*^{RNAi}	359
M, N, O	2	*M1BP*^{RNAi}	289
M, N, O	3	*M1BP*^{RNAi}	452
M, N, O	4	*M1BP*^{RNAi}	159
M, N, O	1	*Cp190*^{RNAi}	551
M, N, O	2	*Cp190*^{RNAi}	466
M, N, O	3	*Cp190*^{RNAi}	460
M, N, O	4	*Cp190*^{RNAi}	183

Supplemental Table 13: *P*-values for Oligopaint FISH experiments for probes G, H, I; probes J, K, L; and probes M, N, O for Figure 8. All knockdowns are indicated in italics. (* $P < 0.05$, ** $P < 0.01$, *** $P < 0.001$).

Panel	Probes	Mock vs.	*P*-value	Significance
c	G-H	$M1BP^{RNAi}$	0.0008	***
c	G-H	$Cp190^{RNAi}$	0.20	ns
c	H-I	$M1BP^{RNAi}$	0.012	*
c	H-I	$Cp190^{RNAi}$	0.61	ns
c	G-I	$M1BP^{RNAi}$	0.018	*
c	G-I	$Cp190^{RNAi}$	0.70	ns
f	J-K	$M1BP^{RNAi}$	0.010	*
f	J-K	$Cp190^{RNAi}$	0.90	ns
f	K-L	$M1BP^{RNAi}$	0.0085	**
f	K-L	$Cp190^{RNAi}$	0.82	ns
f	J-L	$M1BP^{RNAi}$	0.012	*
f	J-L	$Cp190^{RNAi}$	0.68	ns
i	M-N	$M1BP^{RNAi}$	0.0037	**
i	M-N	$Cp190^{RNAi}$	0.84	ns
i	N-O	$M1BP^{RNAi}$	0.0092	**
i	N-O	$Cp190^{RNAi}$	0.73	ns
i	M-O	$M1BP^{RNAi}$	0.0096	**
i	M-O	$Cp190^{RNAi}$	0.91	ns

Supplemental Table 14: Source of ChIP-seq data of other factors.

Factor name	GEO accession
Kc167 CLAMP	GSM2775116
Kc167 BEAF32	GSM762845
S2 ZIPIC	GSM1313421
S2 Pita	GSM1313420
S2 ibf1	GSM1133264
S2 ibf2	GSM1133265
Kc167 CTCF	GSM1535983
