# Supplementary material for: M1BP cooperates with CP190 to activate transcription at TAD borders and promote chromatin insulator activity
Source: Nat Commun. 2021 Jul 7;12:4170. doi: 10.1038/s41467-021-24407-y (PMC8263732; doi:10.1038/s41467-021-24407-y)
Supplement: Supplementary file 3 — Reporting summary [file 41467_2021_24407_MOESM3_ESM.pdf]

nature research

Corresponding author(s): Elissa Lei

Last updated by author(s): Jun 5, 2021

Reporting Summary

Nature Research wishes to improve the reproducibility of the work that we publish. This form provides structure for consistency and transparency in reporting. For further information on Nature Research policies, see our Editorial Policies and the Editorial Policy Checklist.

Statistics

For all statistical analyses, confirm that the following items are present in the figure legend, table legend, main text, or Methods section.

n/a	Confirmed	
☐	☒	The exact sample size (n) for each experimental group/condition, given as a discrete number and unit of measurement
☐	☒	A statement on whether measurements were taken from distinct samples or whether the same sample was measured repeatedly
☐	☒	The statistical test(s) used AND whether they are one- or two-sided<br>*Only common tests should be described solely by name; describe more complex techniques in the Methods section.*
☐	☒	A description of all covariates tested
☐	☒	A description of any assumptions or corrections, such as tests of normality and adjustment for multiple comparisons
☐	☒	A full description of the statistical parameters including central tendency (e.g. means) or other basic estimates (e.g. regression coefficient) AND variation (e.g. standard deviation) or associated estimates of uncertainty (e.g. confidence intervals)
☐	☒	For null hypothesis testing, the test statistic (e.g. F, t, r) with confidence intervals, effect sizes, degrees of freedom and P value noted<br>*Give P values as exact values whenever suitable.*
☒	☐	For Bayesian analysis, information on the choice of priors and Markov chain Monte Carlo settings
☒	☐	For hierarchical and complex designs, identification of the appropriate level for tests and full reporting of outcomes
☐	☒	Estimates of effect sizes (e.g. Cohen's d, Pearson's r), indicating how they were calculated

Our web collection on statistics for biologists contains articles on many of the points above.

Software and code

Policy information about availability of computer code

Data collection	-ChIP-seq libraries were constructed with TruSeq adapters (Illumina) according to the TruSeq Illumina ChIP-seq sample preparation protocol with minor modification as described previously (Bag et al., 2019). All samples were sequenced with HiSeq2500 (Illumina) using 50 bp single-end sequencing at the NIDDK Genomics Core Facility.<br>-The nascent EU-RNA was used to generate RNA-seq libraries with Ovation RNA-seq Systems 1–16 for Model Organisms (Nugen). Samples were sequenced on HiSeq2500 (Illumina) using 50 bp single-end sequencing at the NIDDK Genomics Core Facility.<br>-ATAC-seq was performed following a protocol from the Kaestner Lab (https://www.med.upenn.edu/kaestnerlab/assets/user-content/documents/ATAC-seq%20Protocol%20(Omni)%20-%20Kaestner%20Lab.pdf) with minor modifications. Libraries were constructed using NEBNext HiFi 2x PCR mix (New England Biolabs) and Ad1 and Ad2 primers ((Illumina/Nextera i5 common adapter and i7 index adapters). All samples were sequenced with NextSeq-550 (Illumina) using 50 bp paired-end sequencing at the NIDDK Genomics Core Facility.<br>-TAD border data is downloaded from http://chorogenome.ie-freiburg.mpg.de/.<br>-Images were captured at RT on a Leica DMi 6000B widefield fluorescence microscope using a 1.4 NA 63x or 100x oil-immersion objective and Leica DFC9000 sCMOS Monochrome Camera. DAPI, CY3, CY5, and FITC filter cubes were used for image acquisition. Images were acquired using LasX Premium software (Leica Application Suite X 3.6.0.20104) and deconvolved using Huygens Professional v19.10 software (Scientific Volume Imaging).<br>-For Mass-spectrometry: Proteins were analyzed using tandem HPLC-mass spectrometry at the NIDDK Mass Spectrometry Facility.
Data analysis	ChIP-seq:<br>-FASTQ files of sequenced single-end 50 bp reads were trimmed using cutadapt v1.8.1 (https://cutadapt.readthedocs.io/en/v1.8.1/).<br>-Trimmed reads were mapped to the Flybase r6-24 dm6 genome assembly with Bowtie2 v2.3.5 (https://github.com/BenLangmead/bowtie2).<br>-Multimapping reads were removed mapped reads using samtools v1.9 (https://sourceforge.net/projects/samtools/files/samtools/1.9/).<br>-Duplicates were removed from mapped, uniquely mapping reads with picard MarkDuplicates v2.20.2 (http://broadinstitute.github.io/picard/index.html).

-MACS2 v2.2.5 (https://github.com/taoliu/MACS) was used to call peaks.
-Exons were defined as any exon from any transcript of any gene. Introns were defined as the space between exons derived in a per-transcript manner by using the gffutils v0.10.1 (https://github.com/daler/gffutils).Promoters were defined as the TSS of each transcript plus 1500 bp upstream. Intergenic regions were defined as all regions between gene bodies.
-Binary heatmaps were generated using pybedtools 0.8.1 (https://daler.github.io/pybedtools/).
-Pairwise comparison for co-localization of different factors was performed using the BEDTools 'jaccard' command (Quinlan, A.R., 2010; Dale, R.K., 2011).
-Heat maps were generated by using deepTools packages v3.4.1 in Galaxy.
-Diffbind v2.14.0 /R package using the config object 'data.frame(RunParallel=TRUE, DataType=DBA_DATA_FRAME, AnalysisMethod=DBA_EDGER, bCorPlot=FALSE, bUsePval=FALSE, fragmentSize=300)' and otherwise used defaults (https://bioconductor.org/packages/release/bioc/html/DiffBind.html).
-Motif 1 analysis was derived from the MEME v3.0 analysis (http://meme-suite.org/doc/download.html) of promoters (Ohler, U.,2002). Motif scanning was carried out using FIMO v5.0.1 (Grant, C.E., 2011).
-Fisher's exact tests (FET) were used to test whether the up/down-regulated genes show preference on the overlapped peaks. FET is implemented by Python scipy package v1.5.2 (scipy.stats.fisher_exact, two-sided test).
-Mann-Whitney U test (scipy.stats.mannwhitneyu, two-sided test) for each set of changed genes against unchanged genes are shown. Significant values are corrected by FDR (stats-models.stats.multitest.multipletests).

RNA-seq:
-Adaptors were trimmed from sequence reads using cutadapt v2.7.
-Reads were mapped to the FlyBase r6-24 reference genome using HISAT2 v2.1.0.
-Aligned reads were counted using subread featureCounts v1.6.4
-Differential expression analysis was done with DESeq2 v1.22.1 in v3.5.1 R version.

ATAC-seq:
-FASTQ files of sequenced single-end 51 bp reads were trimmed using cutadapt v1.8.1 (https://cutadapt.readthedocs.io/en/v1.8.1/).
-Trimmed reads were mapped to the Flybase r6-24 dm6 genome assembly with Bowtie2 v2.3.5 (https://github.com/BenLangmead/bowtie2).
-ChrM reads, unmapped reads, non-primary alignment, were removed mapped reads using samtools v1.9 (https://sourceforge.net/projects/samtools/files/samtools/1.9/).
-Duplicates were removed from mapped, uniquely mapping reads with picard MarkDuplicates v2.20.2 (http://broadinstitute.github.io/picard/index.html).
-MACS2 v2.2.5 (https://github.com/taoliu/MACS) was used to call peaks.
-Binary heatmaps were generated using pybedtools 0.8.1 (https://daler.github.io/pybedtools/).
-Pairwise comparison for co-localization of different factors was performed using the BEDTools 'jac-card' command (Quinlan, A.R., 2010; Dale, R.K., 2011).
-Curves were generated by using deepTools v3.4.1 packages in Galaxy.
-Diffbind v2.14.0 /R package using the config object 'data.frame(RunParallel=TRUE, DataType=DBA_DATA_FRAME, AnalysisMethod=DBA_EDGER, bCorPlot=FALSE, bUsePval=FALSE)' and otherwise used defaults (https://bioconductor.org/packages/release/bioc/html/DiffBind.html).
-Fisher's exact tests (FET) were performed using number of gene promoters occupied/not occupied by decreased/increased ATAC-seq peaks from the group of upregulated/downregulated/un-affected gene promoters. FET is implemented by Python scipy package v1.5.2 (scipy.stats.fisher_exact, two-sided test).
-Mann-Whitney U test (scipy.stats.mannwhitneyu) for each set of changed genes against unchanged genes are shown. Significant values are corrected by FDR (statsmodels.stats.multitest.multipletests).

Other:
-Python-3.7.4
-R-4.0.2
-bedtools/2.27.1
-Matplotlib/3.1.1
-Seaborn/0.9.0
-statsmodels/0.12.0
-Scipy/1.5.2
-pybedtools/0.8.1
-Liftover (https://genome.ucsc.edu/cgi-bin/hgLiftOver)

Imaging:
-Images were segmented and measured by a TANGO 3D-segmentation plugin (v 0.99)for ImageJ (v 2.0.0-rc-69/1.53cas) described (Ollion, J., 2013). Hysteresis and Spot Detector 3D are algorithms contained within TANGO.

-Statistical tests were performed using Prism 8 (v8.4.2) software by GraphPad.

Mass-spectrometry:
-Mass from eluted peptides was queried in the UniProt database, and results were analyzed by MaxQuant v1.6.6.0.

For manuscripts utilizing custom algorithms or software that are central to the research but not yet described in published literature, software must be made available to editors and reviewers. We strongly encourage code deposition in a community repository (e.g. GitHub). See the Nature Research guidelines for submitting code & software for further information.

Data

Policy information about availability of data

All manuscripts must include a data availability statement. This statement should provide the following information, where applicable:

- Accession codes, unique identifiers, or web links for publicly available datasets
- A list of figures that have associated raw data
- A description of any restrictions on data availability

The accession numbers for the raw data FASTQ files, processed files, and BigWig files for all sequencing data deposited in NCBI GEO are GSE142533 (https://www.ncbi.nlm.nih.gov/projects/geo/query/acc.cgi?acc=GSE142533) and GSE169105 (https://www.ncbi.nlm.nih.gov/geo/query/acc.cgi?acc=GSE169105).
All other sequencing data for Kc167 CLAMP GSM2775116 (https://www.ncbi.nlm.nih.gov/geo/query/acc.cgi?acc=GSM2775116), Kc167 BEAF32 GSM762845 (https://www.ncbi.nlm.nih.gov/geo/query/acc.cgi?acc=GSM762845), S2 ZIPIC GSM1313421(https://www.ncbi.nlm.nih.gov/geo/query/acc.cgi?acc=GSM1313421), S2 Pita GSM1313420 (https://www.ncbi.nlm.nih.gov/geo/query/acc.cgi?acc=GSM1313420), S2 ibf1 GSM1133264 (https://www.ncbi.nlm.nih.gov/geo/query/acc.cgi?acc=GSM1133264), S2 ibf2 GSM1133265 (https://www.ncbi.nlm.nih.gov/geo/query/acc.cgi?acc=GSM1133265), Kc167 CTCF GSM1535983 (https://www.ncbi.nlm.nih.gov/geo/query/acc.cgi?acc=GSM1535983). Mass spectrometry data files deposited in the PRIDE repository with dataset identifiers PXD026493 (CP190) and PXD026497 [Su(Hw)]. All other relevant data of this study are available from the authors upon request. Additional supplementary data is available in the Supplementary Data file. Source data are provided with this paper.

Field-specific reporting

Please select the one below that is the best fit for your research. If you are not sure, read the appropriate sections before making your selection.

☒ Life sciences ☐ Behavioural & social sciences ☐ Ecological, evolutionary & environmental sciences

For a reference copy of the document with all sections, see nature.com/documents/nr-reporting-summary-flat.pdf

Life sciences study design

All studies must disclose on these points even when the disclosure is negative.

Sample size	Sample sizes were chosen for the assessment of experimental variability. No statistical method was used to predetermine sample size.<br>For enhancer blocking assay we scored 50 flies with clear difference between control and M1BP RNAi flies and barrier activity assay were performed 12 independent larvae from each genotype. For viability assay around 100 animals were analyzed with each Gal4-driver line.<br>For ChIP-seq, we used 2 biological replicates, For RNA-seq, we used 2-3 biological replicates, and ATAC-seq experiments were performed with 3 biological replicates.<br>FISH experiments were performed with 4 biological replicates, and hundreds of cells were examined per condition.<br>See figure legends and supplementary tables for sample size information.
Data exclusions	No data was excluded for analysis.
Replication	Number of replicates for independent experiments are mentioned in figure legends.
Randomization	Our experimental design did not include any randomization of the samples. Samples were grouped according to genotype (wildtype/Control or mutants), or knockdown conditions (Mock or knockdown samples).
Blinding	Investigations were not blinded during data collection as the biological groups (genotypes) were clearly identifiable and handled in parallel. Also, knockdown shows significant knockdown compared to control. Computational analysis was performed by data scientists different from the researchers who collected the data.

Reporting for specific materials, systems and methods

We require information from authors about some types of materials, experimental systems and methods used in many studies. Here, indicate whether each material, system or method listed is relevant to your study. If you are not sure if a list item applies to your research, read the appropriate section before selecting a response.

Materials & experimental systems

n/a	Involved in the study
	[x] Antibodies
	[x] Eukaryotic cell lines
[x]	Palaeontology and archaeology
	[x] Animals and other organisms
[x]	Human research participants
[x]	Clinical data
[x]	Dual use research of concern

Methods

n/a	Involved in the study
	[x] ChIP-seq
[x]	Flow cytometry
[x]	MRI-based neuroimaging

Antibodies

Antibodies used	All antibodies used in the study are described in Supplementary Table 7 for the convenience of the reader.<br>-Guinea pig anti-CP190 (Matzat et al., 2012, Elissa Lei Lab, NIDDK, NIH)<br>-Rabbit anti-CP190 (Moshkovich et al., 2011, Elissa Lei Lab, NIDDK, NIH)<br>-Guinea pig anti-Su(Hw) (Moshkovich and Lei, 2010, Elissa Lei Lab, NIDDK, NIH)<br>-Rabbit anti-Mod(mdg4)67.2 (Van Bortle et al., 2014, Elissa Lei Lab,NIDDK, NIH)<br>-Rabbit anti-M1BP (Li and Gilmour, 2013, David Gilmour Lab, Pennsylvania State University)<br>-Rabbit anti-Pc (Moshkovich et al., 2011, Patrick H. O'Farrell Lab, UCSF)<br>-Mouse anti-Pep (Amero et al., 1991, Ann L. Beyer Lab, University of Virginia School of Medicine)<br>-Rabbit anti-BEAF-32 (Bushey et al., 2009, Victor Corces Lab, Emory University)<br>-Rabbit anti-Histone H3(phospho S10) (Abcam<br>Cat No ab5176)<br>-Mouse anti-Tubulin (Sigma Cat no T6070)
Validation	In WB analysis we observed correct band size according to amino acid length of the protein. For IF we found same binding pattern of proteins.<br>-Guinea pig anti-CP190, Rabbit anti-CP190, Guinea pig anti-Su(Hw), Rabbit anti-Mod(mdg4)67.2, Rabbit anti-M1BP antibodies were validated by Western Blot in the control and the knockdown of the respective protein itself. Guinea pig anti-CP190, Rabbit anti-CP190, Guinea pig anti-Su(Hw), Rabbit anti-Mod(mdg4)67.2 were also validated by immunofluorescence of polytene chromosomes and colocalization with previously validated antibodies raised against the same proteins.<br>-Rabbit anti-Pc was validated by immunostaining in control and mutant Drosophila embryos (Patrick H. O'Farrell Lab)<br>-Mouse anti-Pep was validated by the immunofluorescence assay on polytene staining for visible puffing of the developmentally regulated loci and Western Blotting for specificity by Ann L. Beyer Lab.<br>-Rabbit anti-BEAF-32 was validated by staining polytene chromosomes and showing the absence of staining in mutant flies for specificity (Victor Corces Lab).<br>-Anti-Histone H3(phospho S10) and anti-Tubulin antibodies are commercially available and validated for specificity by western blot by Abcam and Sigma, the respective manufacturers.<br>-Anti-Histone H3(phospho S10) and anti-Tubulin antibodies were co-validated for specificity in our IF assays based on labeling of DNA in mitotic cells and spindle morphology.

Eukaryotic cell lines

Policy information about cell lines

Cell line source(s)	Drosophila melanogaster female embryonic cell line Kc167 obtained from Rachel Green Lab, Johns Hopkins School of Medicine.
Authentication	Cells show close molecular features, morphology, behavior, growth rate and medium requirement as the commercial authorization.
Mycoplasma contamination	Cell lines are tested for Mycoplasma negative.
Commonly misidentified lines (See ICLAC register)	No misidentified cell lines were used in this study.

Animals and other organisms

Policy information about studies involving animals; ARRIVE guidelines recommended for reporting animal research

Laboratory animals	Drosophila melanogaster
Wild animals	This study did not involve wild animals.
Field-collected samples	This study did not involve field-collected samples.

Ethics oversight | N/A

Note that full information on the approval of the study protocol must also be provided in the manuscript.

ChIP-seq

Data deposition

- [x] Confirm that both raw and final processed data have been deposited in a public database such as GEO.
- [x] Confirm that you have deposited or provided access to graph files (e.g. BED files) for the called peaks.

Data access links<br>*May remain private before publication.*	All sequencing data deposited in NCBI GEO are GEO: GSE142533 (https://www.ncbi.nlm.nih.gov/projects/geo/query/acc.cgi?acc=GSE142533). Now all data are available in public.
Files in database submission	Raw data FASTQ files, processed files, and BigWig files for all sequencing data are available with accession numbers.
Genome browser session<br>(e.g. UCSC)	No longer applicable.

Methodology

Replicates: Two replicates per conditions were used for ChIP-seq

Sequencing depth:

ChIP-seq:
-GSM4231396 Kc167_mock_input_R1 Uniquely mapped reads 16803323
-GSM4231397 Kc167_mock_M1BP_R1 Uniquely mapped reads 1628729
-GSM4231398 Kc167_mock_SuHw_R1 Uniquely mapped reads 4191282
-GSM4231399 Kc167_mock_CP190_R1 Uniquely mapped reads 2065932
-GSM4231400 Kc167_mock_MOD_R1 Uniquely mapped reads 2829960
-GSM4231401 Kc167_mock_input_R2 Uniquely mapped reads 12263702
-GSM4231402 Kc167_mock_M1BP_R2 Uniquely mapped reads 1871703
-GSM4231403 Kc167_mock_SuHw_R2 Uniquely mapped reads 3331002
-GSM4231404 Kc167_mock_CP190_R2 Uniquely mapped reads 1978198
-GSM4231405 Kc167_mock_MOD_R2 Uniquely mapped reads 4058756
-GSM4231406 Kc167_M1BPKD_input_R1 Uniquely mapped reads 13544696
-GSM4231407 Kc167_M1BPKD_M1BP_R1 Uniquely mapped reads 5495055
-GSM4231408 Kc167_M1BPKD_SuHw_R1 Uniquely mapped reads 9689870
-GSM4231409 Kc167_M1BPKD_CP190_R1 Uniquely mapped reads 4503589
-GSM4231410 Kc167_M1BPKD_MOD_R1 Uniquely mapped reads 10111078
-GSM4231411 Kc167_M1BPKD_input_R2 Uniquely mapped reads 12400460
-GSM4231412 Kc167_M1BPKD_M1BP_R2 Uniquely mapped reads 2505033
-GSM4231413 Kc167_M1BPKD_SuHw_R2 Uniquely mapped reads 7183423
-GSM4231414 Kc167_M1BPKD_CP190_R2 Uniquely mapped reads 2958819
-GSM4231415 Kc167_M1BPKD_MOD_R2 Uniquely mapped reads 11800598
-GSM4231416 Kc167_mock2_input_R1 Uniquely mapped reads 10981709
-GSM4231417 Kc167_mock2_M1BP_R1 Uniquely mapped reads 3119639
-GSM4231418 Kc167_mock2_CP190_R1 Uniquely mapped reads 3119639
-GSM4231419 Kc167_mock2_input_R2 Uniquely mapped reads 10528940
-GSM4231420 Kc167_mock2_M1BP_R2 Uniquely mapped reads 2118253
-GSM4231421 Kc167_mock2_CP190_R2 Uniquely mapped reads 2870951
-GSM4231422 Kc167_Cp190KD_input_R1 Uniquely mapped reads 3518665
-GSM4231423 Kc167_Cp190KD_CP190_R1 Uniquely mapped reads 4594155
-GSM4231424 Kc167_Cp190KD_M1BP_R1 Uniquely mapped reads 3775439
-GSM4231425 Kc167_Cp190KD_input_R2 Uniquely mapped reads 3886173
-GSM4231426 Kc167_Cp190KD_CP190_R2 Uniquely mapped reads 5185005
-GSM4231427 Kc167_Cp190KD_M1BP_R2 Uniquely mapped reads 3435020
-50 bp single-end sequencing

Antibodies:

-Rabbit anti-CP190 (Moshkovich et al., 2011, Elissa Lei Lab, NIDDK, NIH)
-Guinea pig anti-Su(Hw) (Moshkovich and Lei, 2010, Elissa Lei Lab, NIDDK, NIH)
-Rabbit anti-Mod(mdg4)67.2 (Van Bortle et al., 2014, Elissa Lei Lab, NIDDK, NIH)
-Rabbit anti-M1BP (Li and Gilmour, 2013, David Gilmour Lab, Pennsylvania State University)

Peak calling parameters: MACS2 v2.2.5 (Zhang, Y. et al., 2008) (https://github.com/taoliu/MACS) was used to call peaks by providing replicate IPs and inputs as multiple BAMs, effectively calling peaks on pooled/merged samples and using additional arguments '-f BAM', '--gsize=dm', '--mfold 3 100' (the latter to include a larger set of preliminary peaks for fragment size estimation).

Data quality: -Visual inspection of peaks and inputs tracks.

-Verified differential peaks either in M1BP knockdown and Cp190 knockdown by directed ChIP-qPCR.

Software

-FASTQ files of sequenced single-end 50 bp reads were trimmed using cutadapt v1.8.1 (https://cutadapt.readthedocs.io/en/v1.8.1/).
-Trimmed reads were mapped to the Flybase r6-24 dm6 genome assembly with Bowtie2 v2.3.5 (https://github.com/BenLangmead/bowtie2).
-Multimapping reads were removed mapped reads using samtools v1.9 (https://sourceforge.net/projects/samtools/files/samtools/1.9/).
-Duplicates were removed from mapped, uniquely mapping reads with picard MarkDuplicates v2.20.2 (http://broadinstitute.github.io/picard/ index.html).
-MACS2 v2.2.5 (https://github.com/taoliu/MACS) was used to call peaks.
-Exons were defined as any exon from any transcript of any gene. Introns were defined as the space between exons derived in a per-transcript manner by using the gffutils v0.10.1 (https://github.com/daler/gffutils).Promoters were defined as the TSS of each transcript plus 1500 bp upstream. Intergenic regions were defined as all regions between gene bodies.
-Binary heatmaps were generated using pybedtools 0.8.1 (https://daler.github.io/pybedtools/).
-Pairwise comparison for co-localization of different factors was performed using the BEDTools 'jaccard' command (Quinlan, A.R., 2010; Dale, R.K., 2011).
-Heat maps were generated by using deepTools packages v3.4.1 in Galaxy.
-Diffbind v2.14.0 /R package using the config object 'data.frame(RunParallel=TRUE, DataType=DBA_DATA_FRAME, AnalysisMethod=DBA_EDGER, bCorPlot=FALSE, bUsePval=FALSE, fragmentSize=300)' and otherwise used defaults (https://bioconductor.org/packages/release/bioc/html/DiffBind.html).
-Motif 1 analysis was derived from the MEME v3.0 analysis (http://meme-suite.org/doc/download.html) of promoters (Ohler, U.,2002). Motif scanning was carried out using FIMO v5.0.1 (Grant, C.E., 2011).
-Fisher's exact tests (FET) were used to test whether the up/down-regulated genes show preference on the overlapped peaks. FET is implemented by Python scipy package v1.5.2 (scipy.stats.fisher_exact, two-sided test).
-Mann-Whitney U test (scipy.stats.mannwhitneyu, two-sided test) for each set of changed genes against unchanged genes are shown. Significant values are corrected by FDR (stats-models.stats.multitest.multipletests).
