# Supplementary material for: M1BP cooperates with CP190 to activate transcription at TAD borders and promote chromatin insulator activity
Source: Nat Commun. 2021 Jul 7;12:4170. doi: 10.1038/s41467-021-24407-y (PMC8263732; doi:10.1038/s41467-021-24407-y)
Supplement: Supplementary file 4 — Description of Additional Supplementary Files [file 41467_2021_24407_MOESM4_ESM.pdf]

Description of Additional Supplementary Files

File name: Supplementary Data 1

Description: Enrichment of Motif 1 at +/- 1kb of all TSS at or below *P* = 7.12E-05 analyzed by FIMO (Find Individual Motif Occurrences). The format of this file is described at http://meme-suite.org/doc/fimo-output-format.html.
