# Supplementary material for: M1BP cooperates with CP190 to activate transcription at TAD borders and promote chromatin insulator activity
Source: Nat Commun. 2021 Jul 7;12:4170. doi: 10.1038/s41467-021-24407-y (PMC8263732; doi:10.1038/s41467-021-24407-y)

Source Data 1

Related to Figure 1: Original western blots with size marker indication are shown. Boxes indicate cropped area.

Fig. 1a

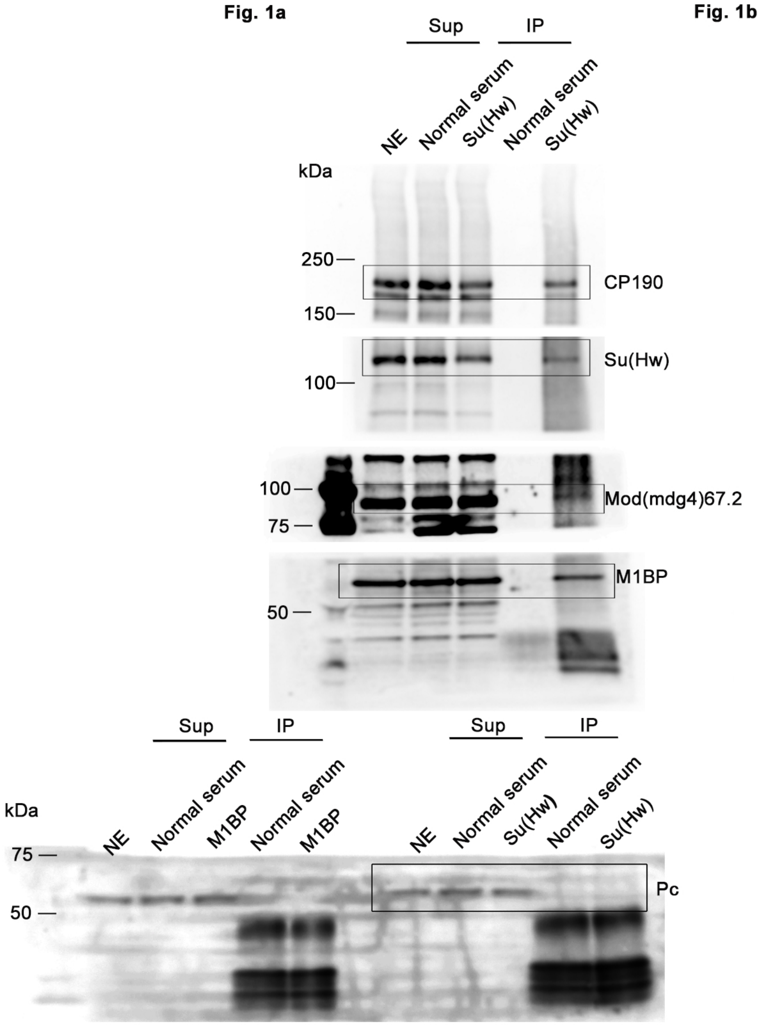

Fig. 1b and 1c

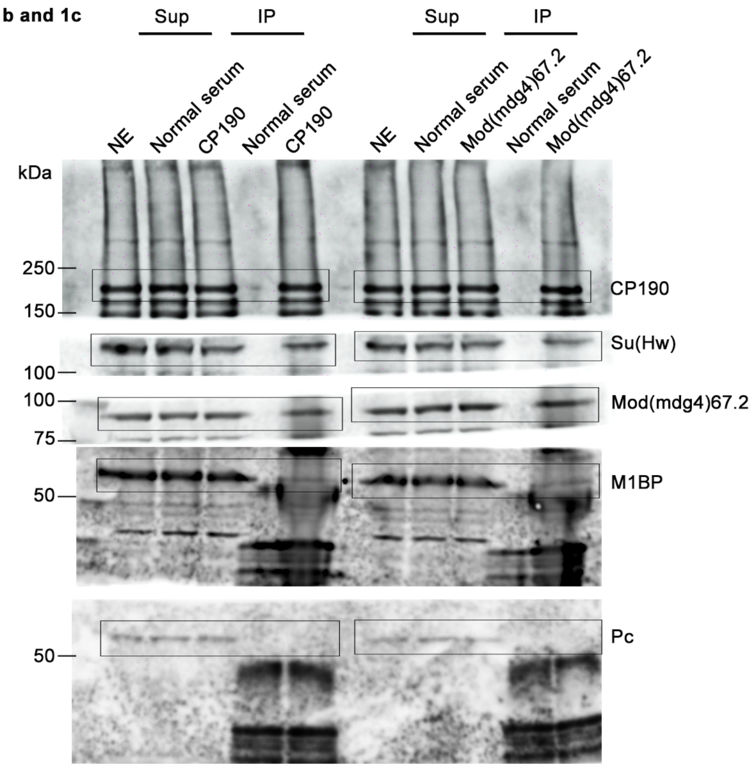

Fig. 1d

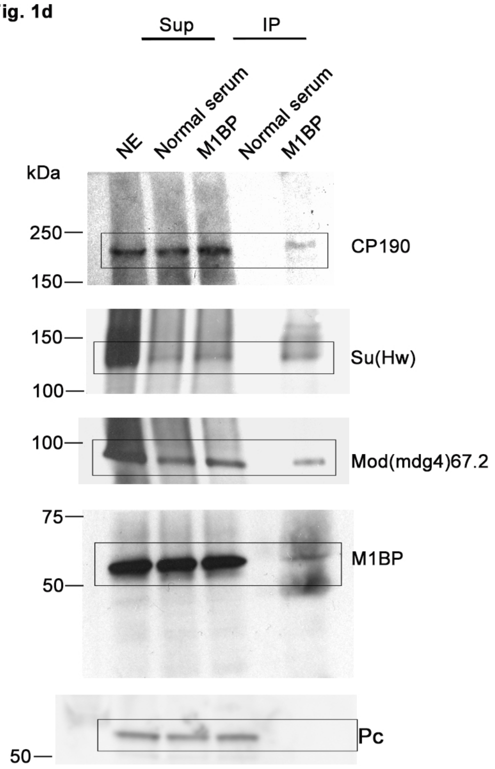

Supplement: Supplementary file 6 — Source Data [file 41467_2021_24407_MOESM6_ESM.zip › Source Data_updated_060121/Source Data 1.pdf]
