# Supplementary material for: M1BP cooperates with CP190 to activate transcription at TAD borders and promote chromatin insulator activity
Source: Nat Commun. 2021 Jul 7;12:4170. doi: 10.1038/s41467-021-24407-y (PMC8263732; doi:10.1038/s41467-021-24407-y)

Source Data 2

Related to Figure 2: Original western blots with size marker indication are shown. Boxes indicate cropped area. Same samples ran on same gel on different lanes for nearby molecular weight of proteins.

Fig. 2a

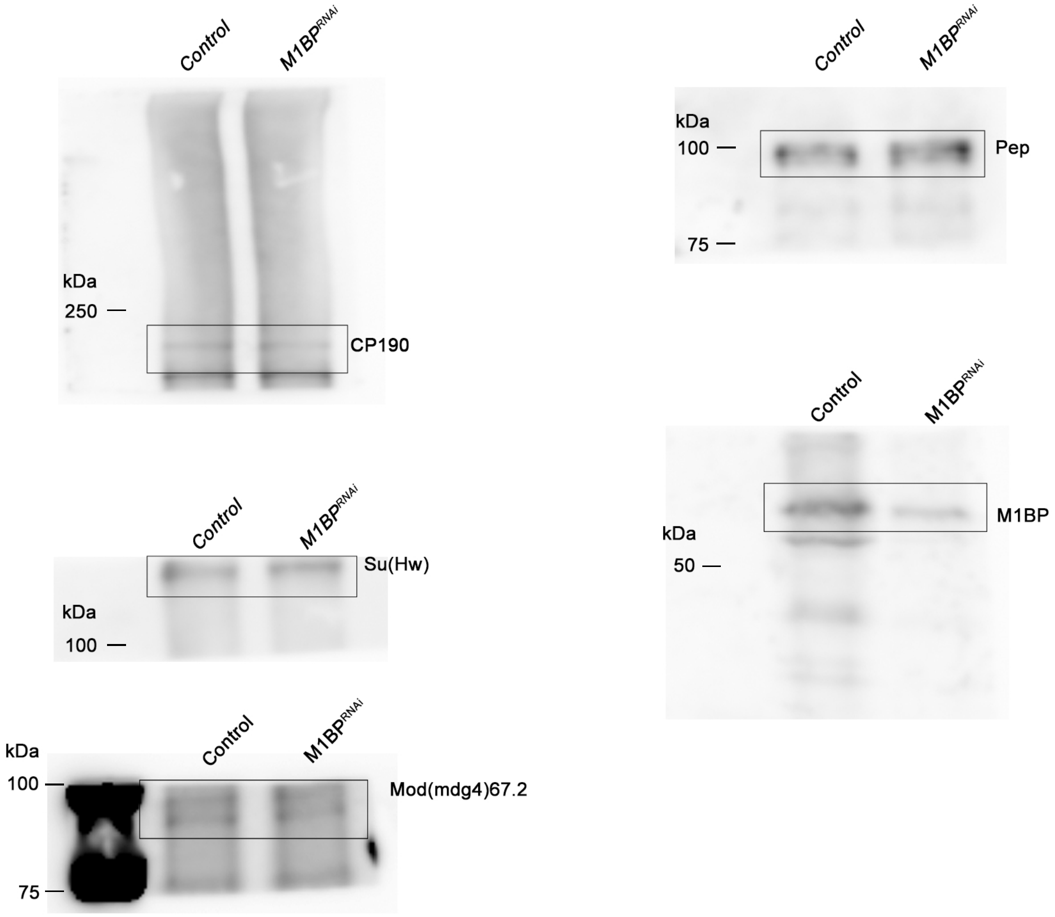

Supplement: Supplementary file 6 — Source Data [file 41467_2021_24407_MOESM6_ESM.zip › Source Data_updated_060121/Source Data 2.pdf]
