# Supplementary material for: M1BP cooperates with CP190 to activate transcription at TAD borders and promote chromatin insulator activity
Source: Nat Commun. 2021 Jul 7;12:4170. doi: 10.1038/s41467-021-24407-y (PMC8263732; doi:10.1038/s41467-021-24407-y)
Supplement: Supplementary file 6 — Source Data [file 41467_2021_24407_MOESM6_ESM.zip › Source Data_updated_060121/Source Data 6.pdf]

Source Data 6

Related to Supplementary Figure 5: Original western blots with size marker indication are shown.
Boxes indicate cropped area.

Supplementary Fig. 5a

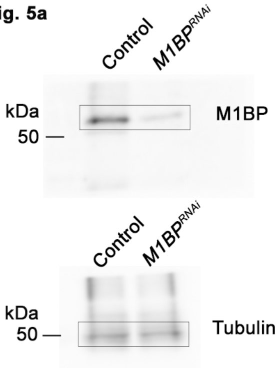

(Due to similar size same protein samples
are loaded in different lanes of same gel)

Supplementary Fig. 5b

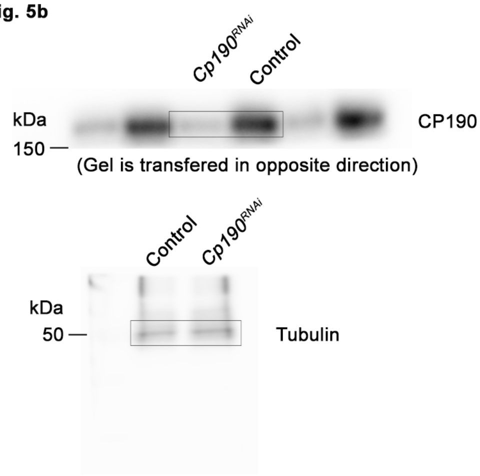

(Same samples are loaded in different gel)

Supplementary Fig. 5c

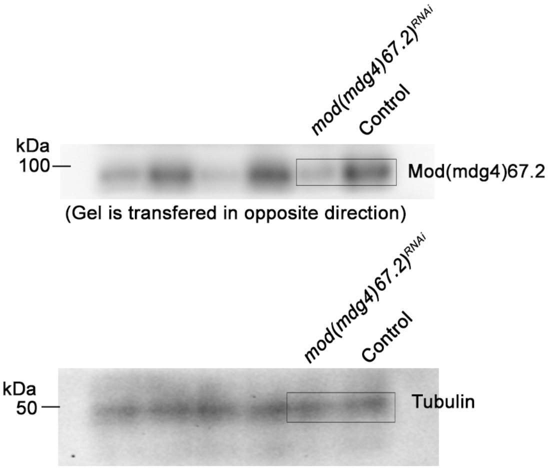

(Same samples are loaded in different gel.
Gel is transfered in opposite direction)
